# Multidimensional insights of electrochemical and quantum investigations of morpholinium cationic surfactants as corrosion inhibitors for carbon steel in acidic solution

**DOI:** 10.1038/s41598-025-05836-x

**Published:** 2025-06-20

**Authors:** A. Elaraby, Doaa F. Seyam, Sherine A. Abdelkader

**Affiliations:** 1 Egyptian Petroleum Research Institute, Nasr City 11727, Cairo, Egypt; 2 Chemistry Department, Faculty of Science, Benha University, Benha, Egypt; 3 Basic Engineering Sciences Department, Faculty of Engineering, Benha University, Benha, Egypt

**Keywords:** Cationic surfactant, Surface tension, EIS, Quantum investigations, Corrosion, Quantum chemistry

## Abstract

Three novel morpholinium-cationic surfactants (coded: DCSM-8, DCSM-10, and DCSM-12) with chemical structure confirmed via FT-IR, HNMR, and mass analysis were applied for carbon steel (*CS*) corrosion control in acidic 4 M HCl solution. The investigated compounds decreased water surface tension (72 mN.m^-1^) to 19.85 mN.m^-1^ after the addition of DCSM-12. The surfactants mitigation performance was assessed via weight loss (*W*_*L*_), potentiodynamic polarization (PDP) and electrochemical impedance spectroscopy (EIS). The synthesized surfactants protected *CS* efficiently with higher inhibition efficiencies up to 97.029% at 1 × 10^–3^ M for DCSM-12 using PDP which also indicated that, the prepared surfactants inhibited both *CS* anodic and cathodic sites with cathodic dominant. EIS data showed higher *CS* resistance from 35.24 Ω.cm^2^ to 1245.54 Ω.cm^2^ after addition of 1 × 10^–3^ M for DCSM-12 with mitigation potency 97.17% which can be attributed to their adsorption process over *CS* surface forming a protective film layer that followed Langmuir adsorption isotherm reflecting the chemical adsorption affinity of the prepared mitigators with higher adsorption energy (ΔG*_ads_) values (> -40 kJ.mol^-1^). Also, the protection effect of the prepared inhibitor (DCSM-12) was confirmed using SEM (scanning electron microscopy) and EDX (energy-dispersive X-ray) showing improvement in *CS* surface morphology. The reactivity of the prepared surfactants and their mitigation role in *CS* deterioration were confirmed theoretically using DFT (density functional theory) and MCs (Monte Carlo simulations).

## Introduction

Sudden failure of materials in several industries is usually caused by acids which are severe to metals^1^. UNS G10300 steel is a medium carbon steel that contains carbon from 0.30% to 2.0% and is characterized by its higher strength, extreme hardness, and wear resistance which make it suitable for manufacturing machinery parts^2–4^. This kind of steel suffers from severe corrosion in acidic solutions or marine environment due to its higher content of pearlite than that in low carbon steel. Pearlite is made up of ferrite and cementite which considered a galvanic microcell inside the structure itself, in the existence of corrosive environment ferrite acts as anode and cementite acts as cathode^5^. Surfactants were used for *CS* protection against corrosion process in acidic medium due to the existence of heteroatoms like Nitrogen, Oxygen, Phosphorous, Sulphur, and/or functionalities with pi electrons which act as adsorption sites onto metal surface film^6–8^. Cationic surfactants are widely used in many applications such as corrosion inhibitors, emulsifiers, wetting agents, and lubricants due to their lower critical micelle concentration, lower surface energy, better physicochemical characteristics and higher solubilization capacity^9–11^. Their higher surface activity form very strong electrostatic bonds with metal surfaces. The main structure of cationic surfactants consists of two positive hydrophilic head linked with hydrophobic chain^12–14^. Most modifications on cationic surfactants have been focused on varying the hydrophobic tail, hydrophilic headgroup, and spacer group in cased of Gemini surfactant by introducing groups such as phenyl or hydrazide, etc. that increase its surface activity and its application as corrosion inhibitors^15–17^. Today, the use of strong acids is an important source of promoting petroleum well productivity. Hydrochloric acid (HCl) is injected into the pipelines to remove the scale layers from their surface. During acid washing, the strong acid causes severe corrosion in the steel pipe wall, reducing the pipe’s strength and resulting in material destruction^18–20^. In economic terms, the huge damage resulted from the corrosion process in petroleum field is more than 3% of the Press Information Bureau (PIB) in the USA. The annual direct cost of metallic corrosion ranges from 2 to 4% of the gross domestic product (GDP) in industrialized countries, and the trend is becoming higher and higher in the future^21–23^.

Guangqiang Xia et al.^24^ evaluated the mitigation power of N-alkyl-3-(2-methoxycarbonyl-vinyl)pyridinium-bromide (MPA-n, n = 8–14) inhibitors using various multi measurements as *W*_*L*_, PDP, EIS, SEM, and EDX showing that, the prepared MPA-n inhibited X70 steel from corrosion in 5 M HCl with inhibition efficiency 96.8% and the inhibition capacity was in the order of MPA-14 > MPA-12 > MPA-10 > MPA-8. Dingli et al.^25^studied the application of two cationic surfactants coded DBBD and QBBD as corrosion mitigators for mild steel in 15% HCl using various measurements including weight loss, electrochemical measurements, scanning kelvin probe (SKP), scanning electron microscope (SEM) and theoretical calculations showing the mitigation power of the prepared mitigators for mild steel corrosion with inhibition potency 95.03 and 97.98 for DBBD and QBBD respectively. M. A. Deyab et al.^26^ investigated the anti-corrosion properties of cationic Gemini surfactant (DMAEB) for N80 C-steel pipe in acid washing solution (15.0% HCl) via gravimetric measurement and potentiodynamic polarization techniques which both confirmed the inhibition performance of the studied mitigators through their adsorption process with mitigation competence efficiency reached 96.8%. A unique kind of conventional surfactant known as a “Cationic Surfactant” has two polar and two non-polar groups that are separated by a spacer^21,22^. This type’s advantages over traditional types include high solubilizing capacity, greatest reduction of surface tension, significant inhibitory efficacy, and low toxicity. Lately various cationic surfactants showed their excellent inhibition against corrosion due to their positive end which is always a quaternary ammonium^27,28^. Cationic surfactants, to the greatest extent of our understanding, have recently been widely used as metal corrosion inhibitors in environment with variable concentrations of HCl, although it has been claimed that these kinds of substances have only sometimes been used in oil field wells^29^.

The novelty in this study is exploring economical, cost-effective, low-toxic, and readily three newly di-cationic surfactants (DCSM-8, DCSM-10, and DCSM-12) based on morpholine containing two polar morpholine heads with ester, hydroxyl and ether groups which facilitate the interaction between the adsorption center of the prepared inhibitors and the empty d-orbital of iron forming a protective barrier layer consequently, mitigate *CS* (UNS G10300) corrosion even at very low concentrations in highly aggressive medium (4 M HCl). In practical applications, it is ideal to provide high protection with a low inhibitor dosage for economical applications. a wide range of concentrations of the inhibitor was used for more comprehensive study starting with lower concentration (1×10^–6^ M) to higher concentration (1×10^–3^ M) showing inhibition efficiency increased till reach 97.029% in the presence of DCSM-12. The studied DCSM-8, DCSM-10, and DCSM-12 with good surface-active parameters were prepared through simple chemical reactions and confirmed using FT-IR,^1^HNMR and Mass spectrum. It is worthwhile to notice that, the prepared cationic surfactants appear to have a good solubility in polar solvents such as water, methanol, ethanol and acetone. The mitigation power of the prepared DCSM-8, DCSM-10, and DCSM-12 was investigated using various techniques as *W*_*L*_, PDP, and EIS besides *CS* surface examination via SEM and EDX were applied. Also, the surface-active parameters of the prepared surfactants showed that, both linked di-functionalized morpholine heads with the accompanying hydrophobic chains have an excellent impact on surface activity and protective properties of the surfactants. Finally, the theoretical quantum chemical investigations reflected the relation between chemical structure of the studied DCSM-8, DCSM-10, and DCSM-12 with their inhibition performance based on DFT and MC_S_.

## Experimental and techniques

### Materials

Dichloromethane (99%, 1.324 g.cm^-3^), nitromethane (98%, 1.130 g.cm^-3^), Morpholine (98.5%, 0.999 g.cm^-3^) and Epichlorohydrin (98.5%, 1.176 g.cm^-3^) were purchased from Loba Chemie, India. Chloroacetic acid (99%, 1.58 g.cm^-3^) was purchased from Piochem, Egypt. octanol, triethylamine, Diethyl ether (99.5%, 0.714 g.cm^-3^), dodecanol (98%, 0.833 g.cm^-3^), hydrochloric acid (35%, 1.18 g.cm^-3^), petroleum ether40-60 (99%, 0.653 g.cm^-3^), ethanol (95%, 0.78 g.cm^-3^), decanol, NaOH (97%, 2.13 g.cm^-3^) and acetone (99.8%, 0.792 g.cm^-3^) were obtained from AL-Nasr Chemicals Co., Egypt. p-Toluene sulfonic acid (98.5%, 1.24 g.cm^-3^), 1,2-dibromoethane (99%, 2.18 g.cm^-3^) and triethylamine (99%, 0.727 g.cm^-3^) were purchased from Sigma-Aldrich, Saint Louis, USA. Also, *CS* (UNS G10300) with a composition of C% 0.28–0.34, Mn% 0.60–0.90, Si% 0.10–0.20, P% 0.04, S% 0.05, Cr% 0.1, Ni% 0.1, Cu% 0.15, N% 0.012, Fe% > 98 (wt. %) was obtained from Suez Steel Company, Suez, Egypt.

### Synthesis of gemini-based morpholinium cationic surfactants

**Fig. 1:**
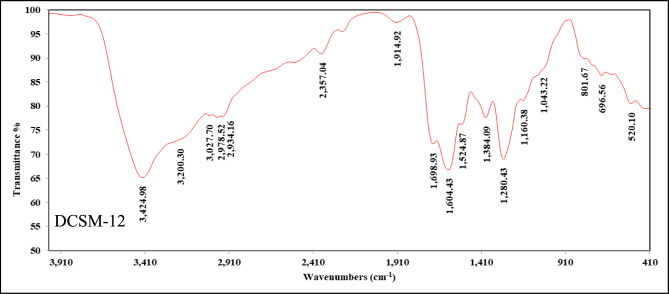
FT-IR of the prepared DCSM-12.

The synthetic route was represented in Fig. 1Si (Supplementary file) as follow:

- Esterification reaction step

Using toluene as a solvent, an esterification reaction was established between. 2-chloroacetic acid and different primary alcohols (octanol, decanol, and dodecanol) in presence of dehydrating agent (p-Toluene sulfonic acid) to obtain three varied ester compounds that were washed with petroleum ether after solvent removal^30^.

- Ether formation step

A mixture of 1 mmol of epichlrohydrin, 1 mmol of primary alcohol (octanol, decanol, or dodecanol) and 1 mmol of sodium hydroxide was refluxed at 313 K for 5 h. Then, the reaction mixture was purified using distilled water and petroleum ether^31^.

- Tertiary amine formation step

The prepared ether compounds (2-((alkoxy) methyl) oxirane) and Morpholine was stirred in molar ratio 1:1 in presence of nitromethane (31 mmol) was at 298 K for 8 h. The mixture was allowed to cool and quenched with water, then extracted with dichloromethane. On the other hand, 1 mmol of Morpholine was refluxed with 1mmol of the obtained ester compounds (alkyl chloroacetate and triethylamine (10 ml) in presence of benzene as a solvent at 80 ⁰C for 6 h. The obtained morpholin-N-ethylalkylate (brown yield) was cooled at room temperature and recrystallized from ethanol^32^.

- Quaternization step

The cationic surfactants (DCSM-8, DCSM-10, and DCSM-12) were prepared by reaction of 1,2-dibromoethane with the obtained two tertiary amines in molar ratio 1:1:1 in dry acetone for 24 h. the reaction mixture was cooled to obtain DCSM-8, DCSM-10, and DCSM-12 as yellow solid precipitate that was washed with acetone and recrystallized with absolute ethanol^33^.

### Surface activity measurements

Surface activity measurements were accomplished with different concentrations of the prepared DCSM-8, DCSM-10, and DCSM-12 at room temperature (298 K) using Tensiometer-K6, KRÜSS, Hamburg, Germany, with a platinum ring in distilled water. The platinum ring was washed distilled water and ethanol to remove any attached particles. Each reading was repeated three times before taking the average final value. Also, all solutions were prepared using double-distilled H_2_O with 72 mN/cm surface tension.

### Weight loss technique (***W***_***L***_)

Using varied emery paper, samples of *CS* were pretreated then washed by a mixture of acetone/ethanol before immersion in corrosive media containing 4 M HCl free and mixed with DCSM-8, DCSM-10, and DCSM-12 at different concentrations (1 × 10^–6^, 1 × 10^–5^, 4 × 10^–5^, 1 × 10^–4^, 4 × 10^–4^, 1 × 10^–3^ M) at room temperature 25 ± 1°C. The corresponding corrosion rate (*r*, mg cm^-2^ h^-1^) and the inhibition efficiency (${\eta }_{{W}_{L}}$) were estimated via the following Eqs.^34^:

1 $$r= \Delta W/At$$

2 $${\eta }_{{W}_{L}}=\frac{{W}_{HCl}-{W}_{i}}{{W}_{HCl}}\times 100$$

where; t is immersion time (h), *A* is *CS* exposed area (cm^2^), and *W*_i_ and *W*_HCl_ represent the mass change with and without inhibitors respectively.

### Electrochemical measurements

Metrohm potentiostat was used for evaluation the mitigation performance of the prepared DCSM-8, DCSM-10, and DCSM-12 via PDP and EIS measurements. Working electrode with surface area 1.0 cm^2^ was connected with platinum electrode as a counter electrode and saturated calomel electrode (SCE) as a reference electrode in 4 M HCl free with and without various concentrations of DCSM-8, DCSM-10, and DCSM-12. Because all kinetics and electrochemical behavior of *CS* is observed fully, PDP curves were presented at large potential window ± 1.0 V around OCP (open circuit potential) using a low scan rate (1 mV.s-^1^)^35,36^. Also, EIS was achieved at frequency range of 100 kHz—0.1 Hz and 10 mV amplitude. The inhibition efficiency was calculated from PDP and EIS based on corrosion current density (*i*_corr_) and polarization resistance (*R*_P_) respectively as follow:

3 $${\eta }_{PDP}= \left(\frac{{\text{i}}_{blank}-{i}_{inh.}}{{i}_{blank}}\right)x 100$$

4 $${\eta }_{EIS}= \left(\frac{{R}_{P.inh.}-{R}_{P.blank.}}{{R}_{P.inh.}}\right)x 100$$

where, *i*_*blank*_ and *i*_*inh.*_ are corrosion current densities for blank solution (4 M HCl) and inhibitors (DCSM-8, DCSM-10, and DCSM-12) respectively. *R*_*P.blank*_ and *R*_*P.inh.*_ are the polarization resistance for *CS* in 4 M HCl free and containing surfactants inhibitors respectively^21^. The standard deviation (*Sd*) values for *R*_P_ and those of *i*_*corr*_ have been calculated and reported in Table 1Si.

### Quantum studies

For more information about the suppression mechanism of the studied inhibitors, theoretical computer–based quantum chemical methods were carried out for determination of the chemical behavior and electron density of DCSM-8, DCSM-10, and DCSM-12 inhibitors based on density fictional theory (DFT). Using Gaussian 09 software, Version Number Rev. C.01, https://gaussian.com/g09_c01/,^37^ supported with GaussView 6, Version Number: 6.0.16, https://gaussian.com/gaussview6/, Semi-mpirical PM6 method was applied for determination DCSM-8, DCSM-10, and DCSM-12 optimized geometry structures then applying B3LYP/6–311 +  + G(d,p) method. The reactivity of the studied inhibitors was discussed by the help of determining the region or sites of HOMO (Highest Occupied Molecular Orbital) and LUMO (Lowest Unoccupied Molecular Orbital) in the inhibitor’s structure. Also, some quantum chemical parameters as electronegativity (χ), the calculated affinity of electron (A), fraction of transferred electrons (∆N), hardness (γ) and the potential of ionization (I) were calculated and discussed. Using, BIOVIA Materials Studio 6.0 (17.1.0.48) software, https://www.3ds.com/products-services/biovia/products/molecular-modeling-simulation/biovia-materials-studio, the adsorption of DCSM-8, DCSM-10, and DCSM-12 inhibitors on Fe (110) plane was simulated using MCs for more evidences about its ability to shield *CS* surface from the corrosive particles and the interaction between the studied inhibitors and Fe (110) in aqueous phase (40 H_2_O). MCs was performed using adsorption locator module involved in BIOVIA Materials Studio 17.1.0.48 software under control COMPASS (Condensed phase Optimized Molecular Potentials for Atomistic Simulation Studies) force field using the optimized structures of the investigated mitigators. The MC annealing process was run in a simulation box of 31.55 × 31.33 × 16.31 Å dimension, and Fe was expanded to a cleavage plan (5 × 5) supercell. Subsequently, a 15-Å vacuum slab was assembled to eliminate the periodic boundary effect over the Fe (110) plane.

### Surface analysis

SEM analysis for *CS* specimens was studied in blank corrosive solution (4 M HCl) with and without 1 × 10^–3^ M of the prepared DCSM-12 after 6 h for more indication about the dissolution behavior of *CS* and DCSM-12 mitigation process using JSM-IT700HR/LA SEM, MA, USA. Besides, EDX unit was used for more data about the outer layer components covered *CS* surface.

## Results and discussion

### Characterization of inhibitors structure

**Fig. 2:**
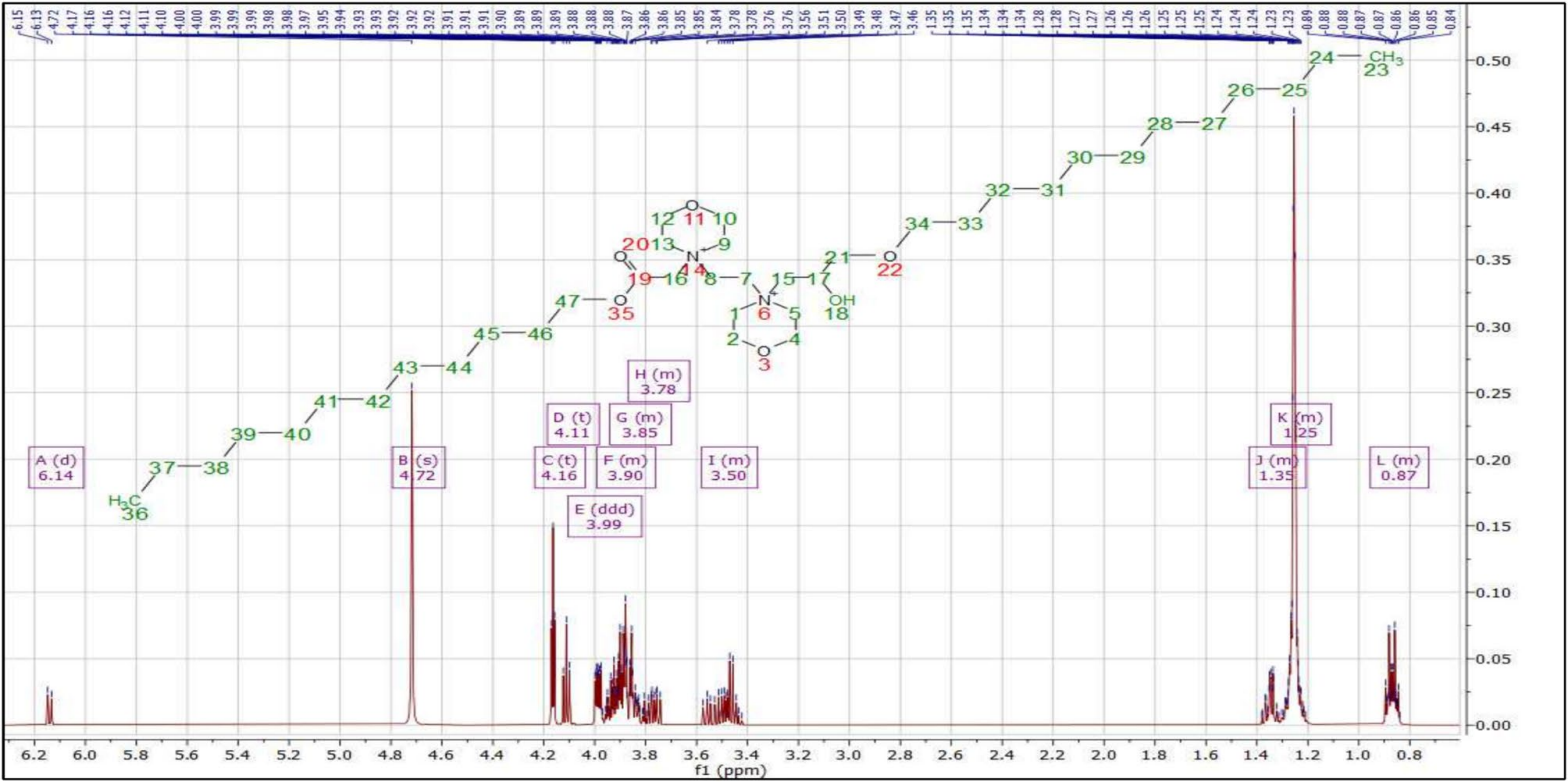
^1^HNMR of the prepared DCSM-12.

The chemical structure of DCSM-8, DCSM-10, and DCSM-12 was confirmed using FT-IR (Thermos Nicolet IS10 FT-IR Spectrophotometer, Thermo Fisher Scientific Inc.), ^1^HNMR (AVANCE III 400 MHz spectrometer, Bruker, Billerica, MA, USA), and Mass (Thermo Scientific, USA, Trace GC Ultra/ISQ Single Quadrupole MS, TG-5MS ) spectrum. Fig. 2Si showed the FT-IR bands of the prepared DCSM-8 at 3439 cm^-1^ (νOH), 3030 cm^-1^ (νCH), 2981, 2935 cm^-1^ (νCH Alkane), 1685 cm^-1^ (νC = O), 1612 cm^-1^ (νCOO), 1397 cm^-1^ (νCH_3_), 1305 cm^-1^ (νCH_2_), 1147 cm^-1^ (νC-O). In case of DCSM-10, the FT-IR (Fig. 2Si) confirmed its chemical structure showing bands at 3414 cm^-1^ (νOH), 3031 cm^-1^ (νCH), 2983, 2938 cm^-1^ (νCH Alkane), 1699 cm^-1^ (νC = O), 1612 cm^-1^ (νCOO), 1424 cm^-1^ (νCH_3_), 1274 cm^-1^ (νCH_2_), 1181 cm^-1^ (νC-O). While, in case of DCSM-12, the FT-IR (Fig. 1) showed bands at 3424 cm^-1^ (ν_OH_), 3027 cm^-1^ (νCH), 2978, 2934 cm^-1^ (νCH Alkane), 1698 cm^-1^ (νC = O), 1604cm^-1^ (νCOO), 1384 cm^-1^ (νCH_3_), 1280 cm^-1^ (νCH_2_), 1160 cm^-1^ (νC-O).

**Fig. 3:**
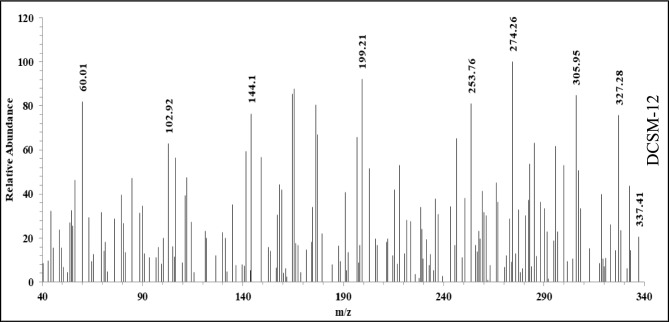
Mass spectra of the prepared DCSM-12.

Fig. 3Si showed the ^1^HNMR of DCSM-8 at 6.12 (m, 1H, 18), 4.72 (s, 1H, 17), 4.16 (m, 2H, 16), 3.88 (m, 2H, 39), 3.85 (m, 8H, 2, 4, 10, 12), 3.82 (m, 4H, 7, 8), 3.80 (m, 2H, 21), 3.78 (m, 2H, 15), 3.48 (m, 8H, 1, 5, 9, 13), 1.35 (m, 6H, 29, 30, 39), 1.26 (m, 22H, 24, 25, 26, 27, 28, 33, 34, 35, 36, 37, 38), 0.86 (m, 6H, 23, 32). In case of DCSM-10, the ^1^HNMR (Fig. 3Si) at 6.11 (m, 1H, 18), 4.87 (s, 1H, 17), 4.14 (m, 2H, 16), 4.00 (m, 2H, 43), 3.89 (m, 8H, 2, 4, 10, 12), 3.86 (m, 4H, 7, 8), 3.83 (m, 2H, 21), 3.80 (m, 2H, 15), 3.77 (m, 8H, 1, 5, 9, 13), 1.61 (m, 6H, 31, 32, 43), 1.26 (m, 30H, 24, 25, 26, 27, 28, 29, 30, 35, 36, 37, 38, 39, 40, 41, 42), 0.88 (m, 6H, 23, 34). While, in case of DCSM-12, the ^1^HNMR (Fig. 2) at 6.21 – 6.08 (d, *J* = 7.5 Hz, 1H, 18), 4.79 – 4.65 (s, 1H, 17), 4.18 – 4.15 (t, *J* = 2.9 Hz, 2H, 16), 4.14 – 4.09 (t, *J* = 6.2 Hz, 2H, 47), 4.01 – 3.97 (ddd, *J* = 7.0, 3.6, 1.9 Hz, 8H, 2, 4, 10, 12), 3.96 – 3.87 (m, 4H, 7, 8), 3.87 – 3.82 (m, 2H, 21), 3.82 – 3.73 (m, 2H, 15), 3.59 – 3.41 (m, 8H, 1, 5, 9, 13), 1.39 – 1.31 (m, 6H, 33, 34, 46), 1.30 – 1.20 (m, 36H, 24, 25, 26, 27, 28, 29, 30, 31, 32, 37, 38, 39, 40, 41, 42, 43, 44, 45), 0.91 – 0.83 (m, 6H, 23, 36).

**Fig. 4:**
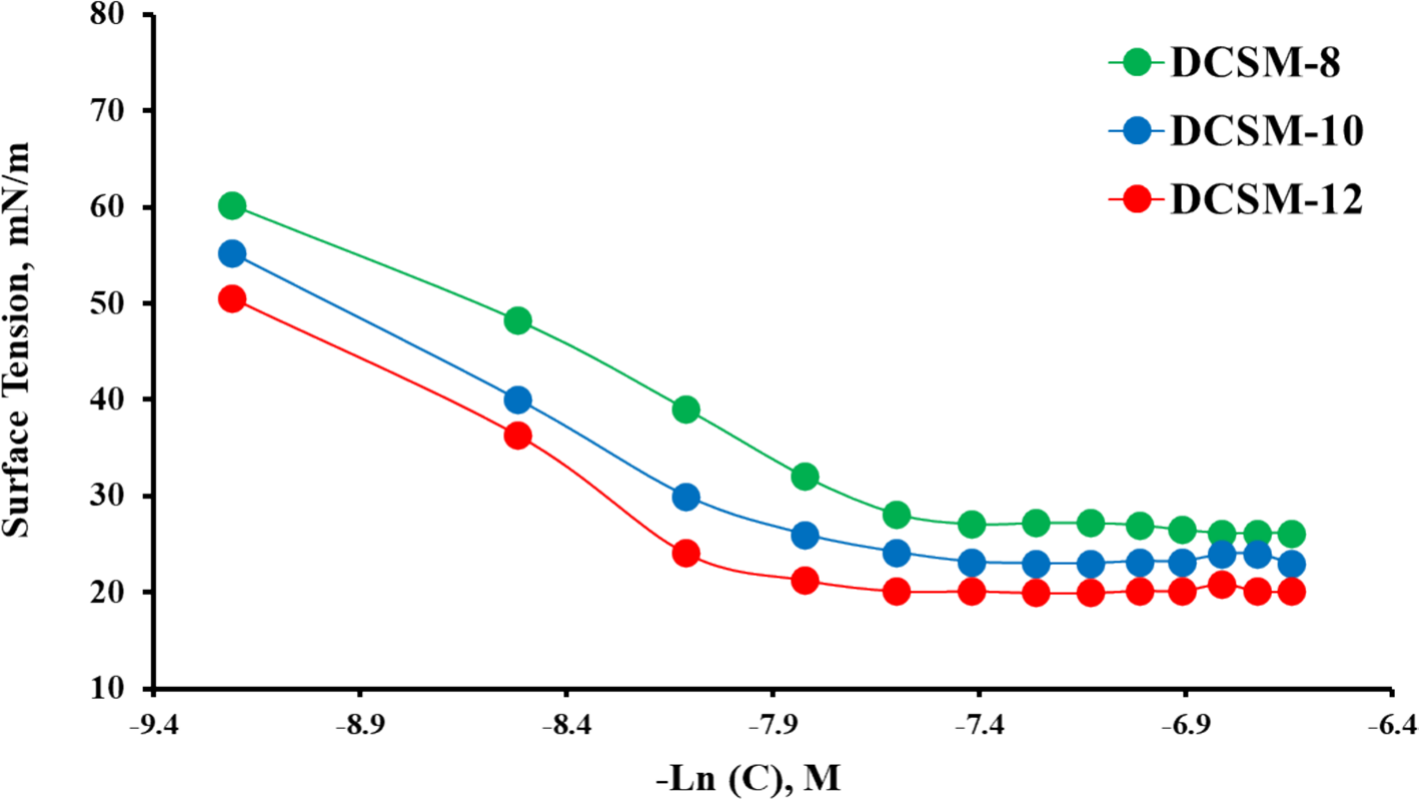
Surface tension variation with surfactants concentration at room temperature.

Fig. 4Si showed the Mass Spectrum (C_31_H_62_N_2_O_6_^2+^) m/z of DCSM-8 with a molecular ion peak at (280.14) m/z: (28.24%), and the Mass Spectrum (C_35_H_70_N_2_O_6_^2+^) m/z of DCSM-10 (Fig. 4Si) with a molecular ion peak at (308.48) m/z: (22.81%). While the Mass Spectrum (C_39_H_78_N_2_O_6_^2+^) m/z of the of DCSM-12 (Fig. 3) with a molecular ion peak at (337.41) m/z: (20.52%).

### Surface-active behavior

The surface activity of the prepared DCSM-8, DCSM-10, and DCSM-12 can be analyzed by measuring surface tension (γ) versus surfactant concentration (-ln) at room temperature (25 °C). Figure 4 showed that, with surfactant concentration rising, a decrease in γ values was observed which can be attributed to the adsorption of surfactant molecules at the air/solution interface. Also, a notable break at (critical micelle concentration) CMC with constant γ values as shown in Fig. 4 can be explained by the saturation occurred at the interface with formation of larger spherical molecules^38,39^. As noticed, DCSM-12 with longer hydrophobic alkyl chain length exhibited the lowest CMC. Some surface-active parameters such as π_**CMC**_ (effectiveness), Maximum surface excess (Γ_ma*x*_), minimum surface area (A_min_), the free Gibbs energy of micellization (${\Delta \text{G}}_{\text{mic}}^{\text{o}}$), and free energy of adsorption (${\Delta G}_{\text{ads}}^{^\circ }$) were calculated and listed in Table.1 as the following equations:

5 $${\pi }_{\text{CMC}}={\gamma }_{\text{o}}- {\gamma }_{\text{CMC}}$$

6 $${\Gamma }_{max}=(-\frac{d{\varvec{\gamma}}}{\mathit{dln}C})/\left(2.303nRT\right)$$

7 $$A_{\min } = {\raise0.7ex\hbox{${10^{14} }$} \!\mathord{\left/ {\vphantom {{10^{14} } {N_{A} \Gamma_{MAX} }}}\right.\kern-0pt} \!\lower0.7ex\hbox{${N_{A} \Gamma_{MAX} }$}}$$

8 $${\Delta G}_{mic}^{o}=2.303RT\text{log}CMC$$

9 $$\Delta G_{ads}^{^\circ } = \Delta G_{mic}^{^\circ } - \left( {0.06023 \times {\raise0.7ex\hbox{${\pi_{CMC} }$} \!\mathord{\left/ {\vphantom {{\pi_{CMC} } {\Gamma_{\max } }}}\right.\kern-0pt} \!\lower0.7ex\hbox{${\Gamma_{\max } }$}}} \right)$$

where n, *R*, *T* and *N*_A_ are, number of ions dissociation (n = 3), absolute temperature (K) and Avogadro’s number^29^. As noticed in Table 1, DCSM-12 with highest π_**CMC**_ value indicating its ability to decrease surface tension compared with the other surfactants consequently its high inhibition efficacy^40,41^. Also, the drops in ${\Gamma }_{max}$ accompanied with ${A}_{min}$ enhancement as shown in Table 1, indicating the role hydrophobic chain in large surface area occupation^42^. The values of ${\Delta G}_{mic}^{^\circ }$ and ${\Delta G}_{\text{ads}}^{^\circ }$ with -ve sign reflecting the spontaneous process of micellization and adsorption. The calculated value of ${\Delta G}_{\text{ads}}^{^\circ }$ relative to that of ${\Delta G}_{mic}^{^\circ }$ exhibited that the prepared surfactants favored adsorption than micellization which confirmed that their higher mitigation power^43,44^.

**Table 1:** Surface active parameters of the prepared surfactants at room temperature.

Surfactant	CMC, M × 10^–4^	*γ*_CMC_, mN/m	*π*_CMC_, mN/m	*Γ*_max_ × 10^10^, mol.cm^-2^	*A*_min_, nm^2^	∆G^o^_mic_, kJ/mol	∆G^o^_ads_, kJ/mol
DCSM-8	5.325	27.92	44.08	3.94	0.422	-28.62	-39.82
DCSM-10	5.247	22.74	49.26	3.73	0.446	-28.66	-41.88
DCSM-12	4.921	19.85	52.15	3.64	0.456	-28.82	-43.15

### Electrochemical methods

#### Potentiodynamic polarization (PDP)

**Fig. 5:**
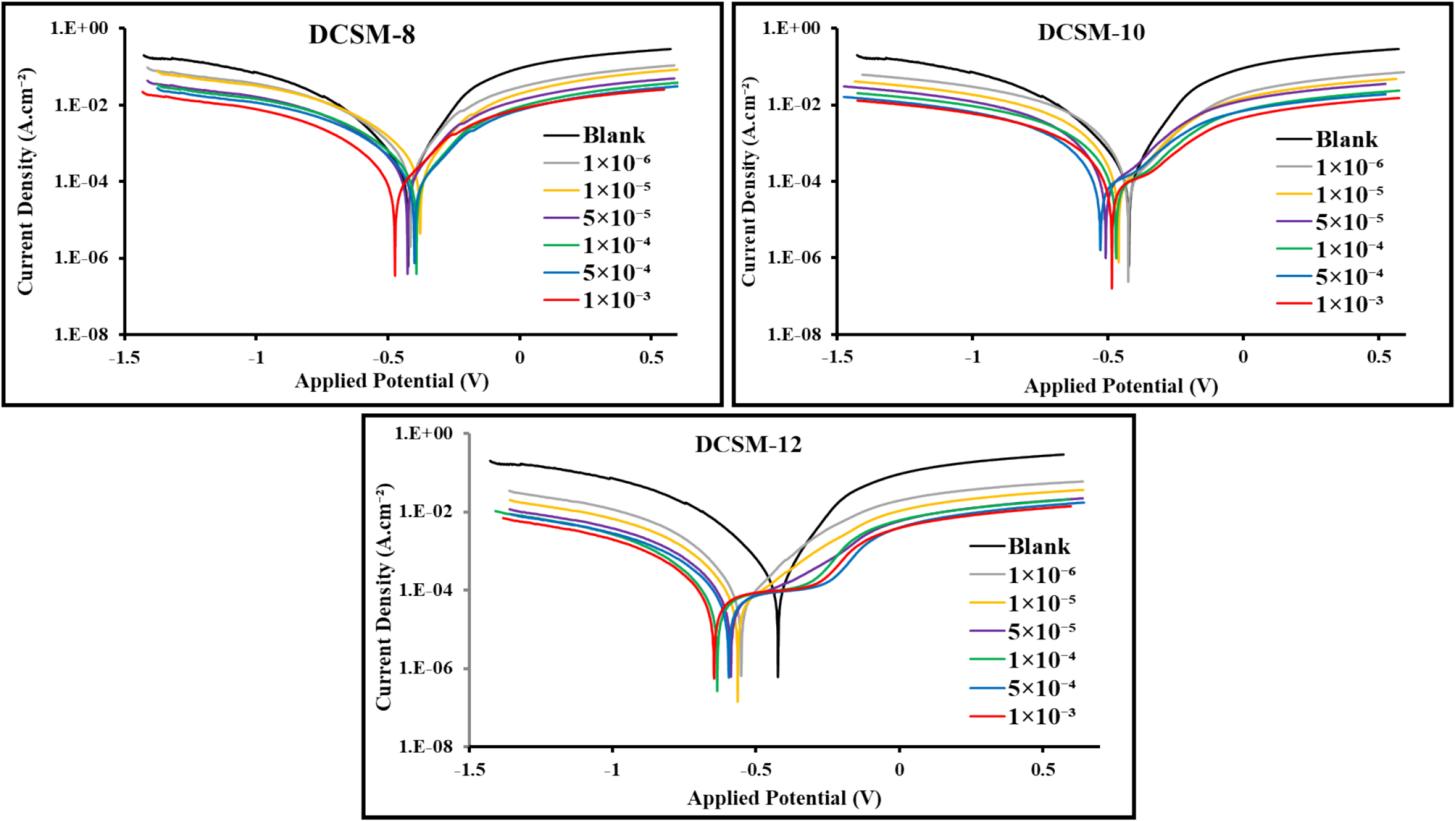
PDP curves for *CS* in the absence and presence of DCSM-8, DCSM-10, and DCSM-12 inhibitors at room temperature.

Tafel curves of *CS* reaction in 4 M HCl free in absence and presence of several doses of DCSM-8, DCSM-10, and DCSM-12 inhibitors were represented as shown in Fig. 5 The similarity of PDP curves of *CS* with and without several doses of the studied surfactants confirmed that, the corrosion reaction mechanism of *CS* did not change. Also, the introducing of DCSM-8, DCSM-10, and DCSM-12 inhibitors to the corrosive solution shifted PDP curves to less active areas confirming their mitigation role^45^. The gap between the PDP curves of the untreated solution.

and those of DCSM-8, DCSM-10, and DCSM-12 inhibitors enhanced with increasing their.

concentration owing to the adsorption route pointing that the investigated surfactants inhibit the *CS* corrosion process^46,47^. The parallel shape of cathodic lines and their shift to lower current value as in Fig. 5 exhibited that, *CS* corrosion mechanism and H_2_ evolution reaction not affected by inhibitors addition^48–50^. This shift increases with rising of inhibitors doses due to number rising of DCSM-8, DCSM-10, and DCSM-12 adsorbed molecules that cover extra *CS* surface area and consequently limits its dissolution rate and retards H_2_ evolution mechanism (Volmer-Heyrovsky) via blocking the corrosion reactive sites at *CS* surface by DCSM-8, DCSM-10, and DCSM-12 adsorbed molecules which decrease the available surface area for H^+^ ions adsorption and reduction. So, the corrosion reaction mechanism remains unaffected^22,51^. As in Fig. 5, the addition of the prepared DCSM-8 shifted PDP to both anodic and cathodic regions which confirming its mixed-type of inhibition while a notable shift in PDP curves to -ve direction after the addition of DCSM-10, and DCSM-12 can be explained by more number of DCSM-10, and DCSM-12 molecules adsorbed on *CS* surface and block *CS* cathodic regions more than its anodic regions^52,53^. In addition, anodic Tafel lines give an indication about role of the studied inhibitors in *CS* corrosion control which can be detected as modification in anodic line slope with inhibitors concentrations via their adsorption process forming an insulation barrier layer over *CS* surface^54,55^. Also, some corrosion parameters as anodic and cathodic Tafel slopes (*β*_a_ and* β*_c_) and corrosion potential (*E*_corr_) were extracted and tabulated as in Table 2. From Table 2, the values of *E*_corr_, *β*_a_ and *β*_c_ reflected that of DCSM-8, and DCSM-10 acted as mixed-type inhibitor while DCSM-12 acted as cathodic type inhibitor^56,57^. The drops in *i*_corr_ value after DCSM-8, DCSM-10, and DCSM-12 addition can be explained by their blocking effect for both cathodic and anodic sites of *CS* surface^58,59^. The calculated value of ${\eta }_{PDP}$ confirmed that, the prepared mitigators protect *CS* effectively in corrosive 4 M HCl through their adsorption process by shielding its surface via construction of defensive layer against the destructive species^60^. As in Table 2, the value of ${\eta }_{PDP}$ enhanced till touch 93.966%, 94.453%, and 97.029% at 1 × 10^–3^ M of DCSM-8, DCSM-10, and DCSM-12 respectively. All these annotations powered the adsorption behavior of the prepared inhibitors and their role in *CS* protection. The value of ${\eta }_{PDP}$ increases in the order DCSM-12 > DCSM-10 > DCSM-8 which can be explained by the presence of longer hydrophobic chain in DCSM-12 structure which enhanced its mitigation efficacy for *CS* in 4 M HCl electrolyte.

**Table 2:** PDP data for *CS* in 4 M HCl free and containing various concentrations of the prepared inhibitors at room temperature.

*Inh*	Conc. M	*β*_*a*_ (mV/dec)	*-β*_*c*_ (mV/dec)	-*E*_corr_ (mV)	*i*_corr_ (mA)	*r* (mm/year)	*θ*	${\eta }_{PDP}$
–	Blank	141.90	224.80	440.55	23.60	274.18	–	–
DCSM-8	1 × 10^–6^	179.30	255.20	432.92	2.812	32.82	0.88084	88.084
	1 × 10^–5^	180.40	254.20	432.61	2.394	27.73	0.89855	89.855
	5 × 10^–5^	181.90	252.50	476.21	2.057	23.66	0.91283	91.28
	1 × 10^–4^	182.10	250.90	448.66	1.891	21.95	0.91987	91.987
	5 × 10^–4^	183.50	249.30	429.72	1.684	19.85	0.92864	92.864
	1 × 10^–3^	176.10	235.50	451.07	1.424	16.88	0.93966	93.966
DCSM-10	1 × 10^–6^	138.80	240.20	442.04	2.727	31.42	0.88444	88.444
	1 × 10^–5^	138.70	239.00	498.82	2.254	26.25	0.90449	90.449
	5 × 10^–5^	140.60	236.40	459.02	2.002	22.68	0.91516	91.516
	1 × 10^–4^	144.50	234.90	440.22	1.791	20.49	0.9241	92.411
	5 × 10^–4^	144.90	232.10	507.36	1.558	18.08	0.93398	93.398
	1 × 10^–3^	145.40	238.90	496.52	1.309	15.27	0.94453	94.453
DCSM-12	1 × 10^–6^	199.80	270.80	543.64	2.286	26.74	0.90313	90.313
	1 × 10^–5^	149.20	267.00	536.99	2.041	23.53	0.91351	91.351
	5 × 10^–5^	170.40	250.30	558.99	1.595	18.26	0.93241	93.241
	1 × 10^–4^	120.20	279.40	553.87	1.112	12.78	0.95288	95.288
	5 × 10^–4^	121.00	256.90	560.76	0.902	10.46	0.96178	96.177
	1 × 10^–3^	155.40	273.00	570.52	0.701	8.13	0.97029	97.029

#### Electrochemical impedance spectroscopy

**Fig. 6:**
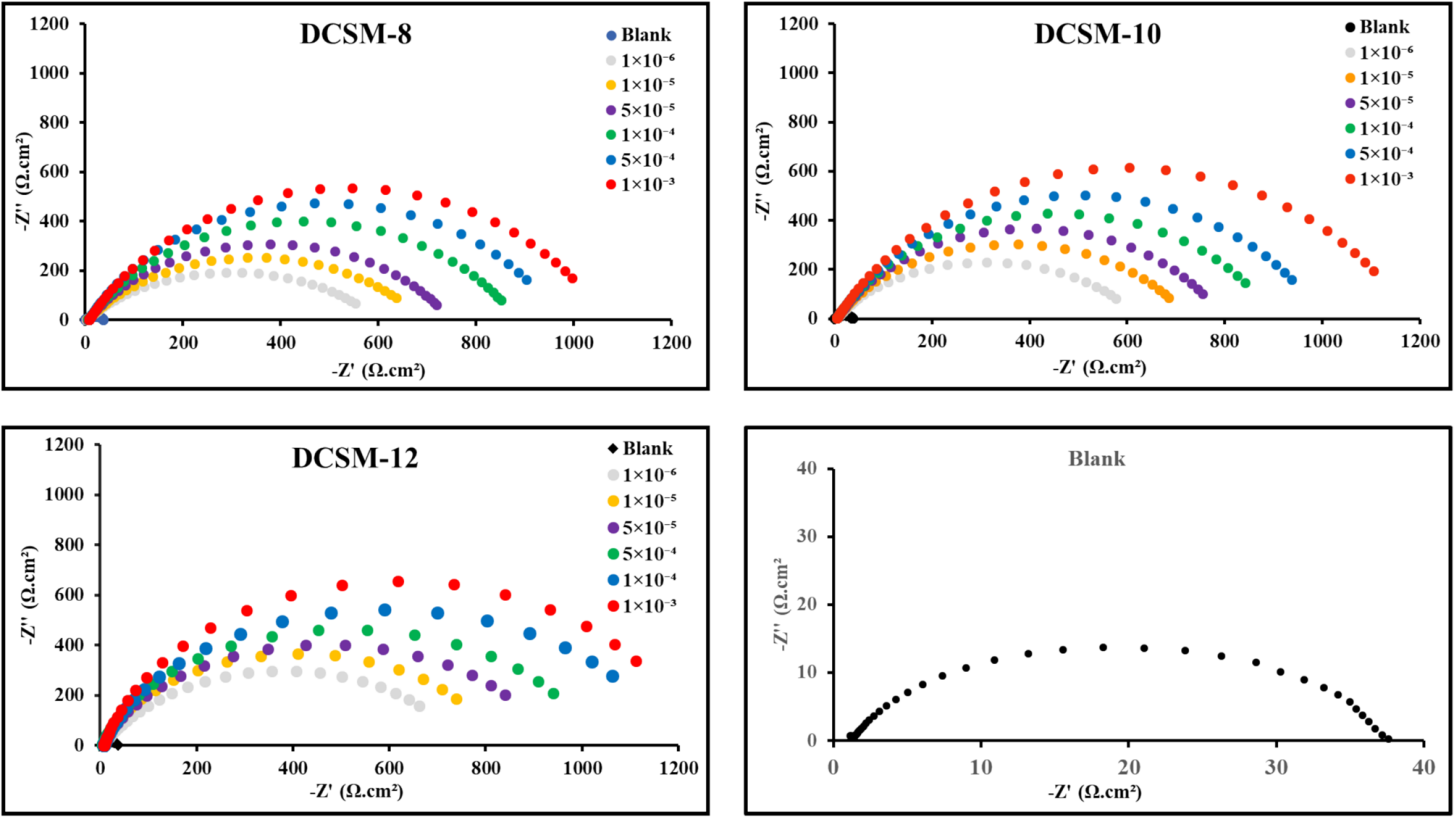
Nyquist plots for *CS* in 4 M HCl with and without various concentrations of the prepared surfactants. at room temperature.

**Fig. 7:**
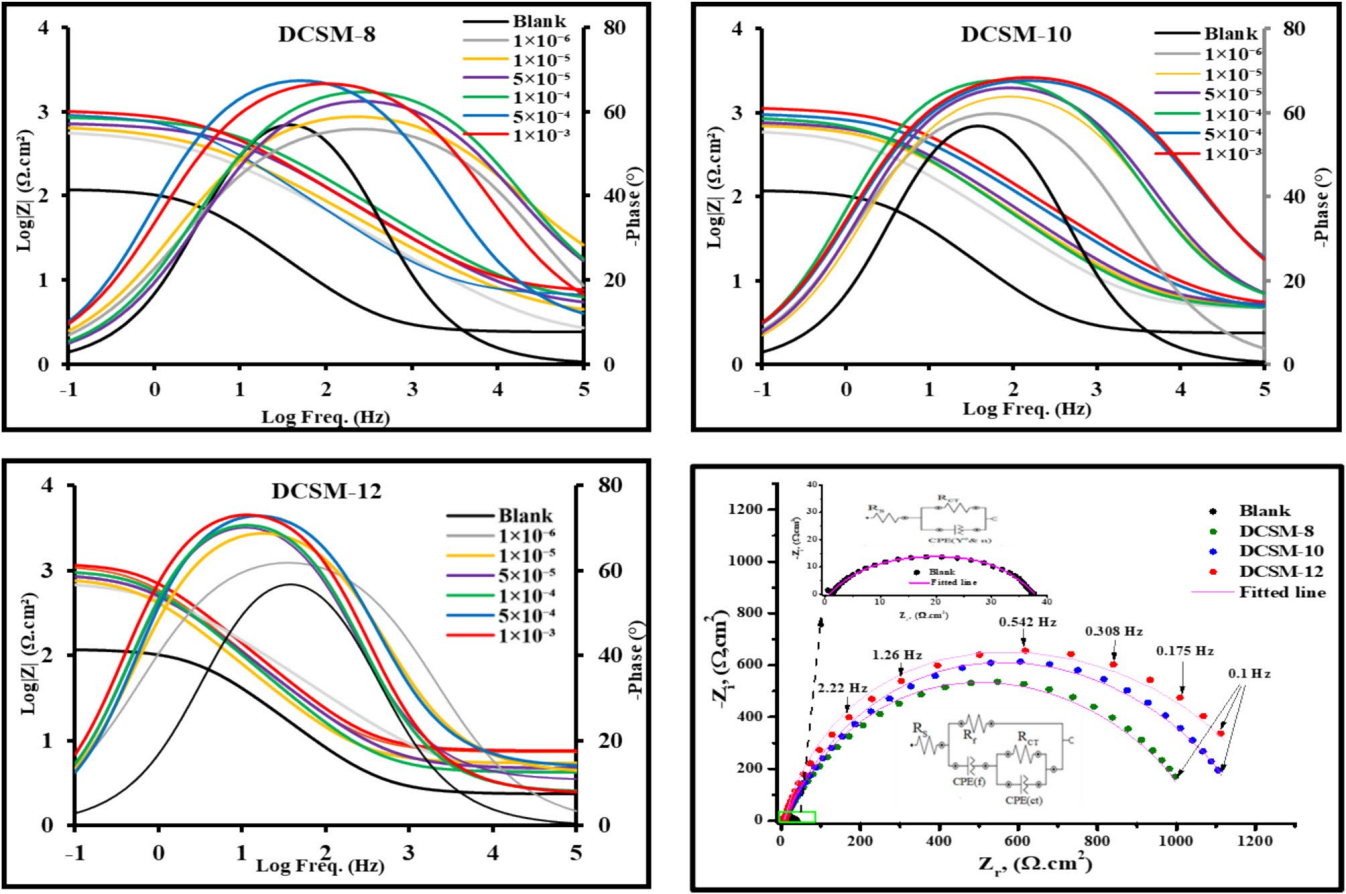
Bode-Phase diagrams of *CS* in absence and presence of different concentrations of the prepared DCSM-8, DCSM-10, and DCSM-12 inhibitors at room temperature and the proposed equivalent circuit of *CS* with and without1 × 10^–3^ M of the prepared surfactants.

It is imperative to achieve a steady state process before starting electrochemical measurements in order to establish *CS* behavior. *E*_ocp_ variation of *CS* with time in the inhibited and uninhibited solutions until the steady-state potential was depicted as seen in Fig. 5Si. It was noticed that, OCP tends to be stable with time and less fluctuates were observed. Also, the rapidly and negligible variations in the OCP may be attributed to the modification of the interface surface at the studied inhibitors doses with a steady-state had been achieved after 5 min. This result suggested that, the studied DCSM-8, DCSM-10, and DCSM-12 have more thermodynamically stable state and effective adsorption over *CS* surface^21,61^. Also, the addition of investigated inhibitors shifted* E*_ocp_ value to negative (cathodic) direction which can be explained by the adsorption process of the prepared inhibitors over *CS* surface and dominate the cathodic reaction more than anodic reaction^62,63^. EIS response of *CS* was presented as in Fig. 6 and Fig. 7 showing *CS* Nyquist plots and Bode-Phase curves respectively in acidic solutions (4 M HCl) with and without various concentration of the studied surfactants (DCSM-8, DCSM-10, and DCSM-12). As noticed, the diameter of Nyquist semicircle increases with surfactants doses rising which powered the adsorption capacity of the prepared inhibitors over *CS* surface and their inhibition performance via construction of an insulation layer over *CS* surface which blocks the ionization of *CS* that can be confirmed as in Nyquist diameter rising to higher values^64,65^.Also, the similarity of Nyquist curves in the inhibited and uninhibited solutions as in Fig. 6 indicated that, the corrosion process was governed by charge transfer process, while their imperfect semicircles shapes can be clarified with roughness and inhomogeneity of *CS* surface^66,67^. The addition of DCSM-8, DCSM-10, and DCSM-12 inhibitors shifted Phase-curves towards -90 and Bode-curves to higher value which exhibited their adsorption power over *CS* surface and formation of a shielding layer against the destructive attack of the acidic liquid^68^. The proposed equivalent circuit (EC) of *CS* in Fig. 7 for 4 M HCl free consisted of *R*_s_ (solution resistance), *CPE* (constant phase element) which can be defined by *Y*^o^ and coefficient *n* and *R*_p_ (polarization resistance) which involved *R*_ct_ (charge transfer resistance)*, R*_d_ (diffuse layer resistance) and *R*_a_ (accumulation resistance)^69^, while after the addition of the prepared surfactants, EC with two time constant comprised of *R*_s_, *CPE* and *R*_p_ (= *R*_ct_ + *R*_d_, *R*_a_ and *R*_f_ “film resistance”) was detected indicating that, the corrosion mechanism of *CS* changed due to stable film formation^70–72^. *n* value as tabulated in Table 3 declined with the addition of DCSM-8, DCSM-10, and DCSM-12 indicating that, *CS* surface become more homogeneous after the addition of surfactants owing to their adsorption over *CS*^73,74^. The lowering in *n* value can be explained by irregular current distribution arises from *CS* roughness and defects of its surface besides the diameter’s variance between electrons and inhibitors molecules, as electrons dominance charges on *CS* side of *CS*/HCl interface, while inhibitors adsorbed molecules dominance charges on HCl solution side. The overall charges of electrons (-ve) and inhibitors adsorbed molecules (+ ve) on *CS* surface are equal. Also, the n value < 1 is due to *CS*/solution interface not behaving as an ideal capacitor^60,75,76^. Also, Y^o^ values decreased with DCSM-8, DCSM-10, and DCSM-12 addition which can be attributed to corrosive particles replacement process from *CS* surface with the adsorbed inhibitors molecules consequently, the film thickness (*T*) over *CS* surface increased^1,4^. The adsorbed inhibitors layer can change metal interfacial structure. According to Helmholtz equation, the double layer capacitance (*C*_dl_) value decreased with DCSM-8, DCSM-10, and DCSM-12 addition as the following Eq.^77^:

10 $${C}_{\text{dl}}=\left(\frac{{\varepsilon }^{o}\varepsilon }{T}\right)A$$

11 $${C}_{\text{dl}}=1/(2\uppi {R}_{P}{F}_{img\to Max})$$

12 $$\uptau ={C}_{\text{dl}}\times {R}_{\text{ct}}$$

here, ε◦ and ε are air permittivity and local dielectric constant. *A* means angular frequency and *F*_*img → Max*_ denotes maximum frequency at Z_i_ (imaginary resistance)^78–80^. From Table 3, it was observed that, the drop in *C*_dl_ value with rising of DCSM-8, DCSM-10, and DCSM-12 doses can be explained by the replacement of the destructive particles on *CS* surface by mitigators adsorbed molecules consequently the film thickness over *CS* surface increased with concentration which was also confirmed from the obtained Y^o^^81,82^. All these observations exhibited the mitigation power of the studied inhibitors for *CS* against the corrosive surrounding^83,84^. Also, the time related to charge distribution equilibrium after an electric disturbance is called $\tau$ (relaxation time). The existence of DCSM-8, DCSM-10, and DCSM-12 shifted $\tau$ value to higher value which reflected the adsorption time process becomes much higher which denotes.

**Table 3:** Impedance parameters for *CS* in 4 M HCl free and containing various concentrations of the prepared inhibitors at room temperature.

*Inh*	Conc. M	R_S_ (Ω.cm^2^)	CPE		R_ct_ (Ω.cm^2^)	R_f_ (Ω.cm^2^)	R_P_ (Ω.cm^2^)	*C*_dl_, (F/cm^2^) × 10^–9^	*τ*, (s)	χ^2^ × 10^–3^	${\eta }_{EIS}$
			n	Y° (μF)							
–	Blank	2.386 ± 1.02	0.8532 ± 0.07	418.18 ± 1.45	35.24 ± 3.13	–	35.24 ± 1.15	65.81	0.0134	4.013	–
DCSM-8	1 × 10^–6^	2.707 ± 0.91	0.8525 ± 0.08	62.29 ± 1.27	545.53 ± 2.05	8.24 ± 0.15	553.77 ± 1.06	9.75	0.030	3.241	93.64
	1 × 10^–5^	4.488 ± 0.80	0.8488 ± 0.07	22.57 ± 1.35	628.64 ± 3.14	9.31 ± 0.09	637.95 ± 1.33	8.46	0.038	2.847	94.48
	5 × 10^–5^	5.419 ± 0.72	0.8379 ± 0.03	17.33 ± 2.02	704.24 ± 3.42	12.52 ± 0.08	716.76 ± 2.20	6.28	0.050	2.771	95.08
	1 × 10^–4^	6.230 ± 1.13	0.8303 ± 0.09	13.53 ± 1.93	832.3 ± 4.06	16.33 ± 0.08	848.63 ± 1.13	3.62	0.058	1.984	95.85
	5 × 10^–4^	6.809 ± 0.92	0.8232 ± 0.08	11.39 ± 2.28	891.47 ± 2.65	19.84 ± 0.14	911.31 ± 3.05	3.04	0.064	0.859	96.13
	1 × 10^–3^	7.687 ± 0.85	0.8095 ± 0.06	8.25 ± 3.30	982.88 ± 4.12	21.27 ± 0.17	1004.15 ± 2.17	1.63	0.072	1.221	96.49
DCSM-10	1 × 10^–6^	4.702 ± 1.14	0.8265 ± 0.12	49.48 ± 2.15	593.32 ± 1.55	9.11 ± 0.07	602.43 ± 2.21	7.57	0.071	2.230	94.15
	1 × 10^–5^	4.915 ± 0.76	0.8367 ± 0.08	18.24 ± 2.34	684.48 ± 1.96	11.34 ± 0.14	695.82 ± 2.50	7.02	0.083	2.182	94.94
	5 × 10^–5^	5.296 ± 0.90	0.8452 ± 0.16	16.08 ± 1.86	768.54 ± 2.77	17.02 ± 0.09	785.56 ± 1.37	6.11	0.088	1.873	95.51
	1 × 10^–4^	4.805 ± 1.04	0.8388 ± 0.09	10.43 ± 3.10	872.42 ± 1.69	20.16 ± 0.19	892.58 ± 2.26	3.06	0.091	1.624	96.05
	5 × 10^–4^	4.937 ± 0.82	0.8456 ± 0.09	8.86 ± 1.79	963.72 ± 2.60	24.41 ± 0.16	988.13 ± 3.09	2.24	0.094	0.937	96.43
	1 × 10^–3^	5.602 ± 0.68	0.8456 ± 0.08	7.10 ± 2.46	1087.59 ± 3.15	28.97 ± 0.08	1116.56 ± 1.14	1.41	0.103	0.881	96.84
DCSM-12	1 × 10^–6^	4.209 ± 0.87	0.8234 ± 0.06	21.17 ± 3.06	676.75 ± 2.51	10.28 ± 0.07	687.03 ± 3.05	4.42	0.166	2.024	94.87
	1 × 10^–5^	5.413 ± 0.96	0.8345 ± 0.03	13.69 ± 3.44	788.73 ± 3.20	13.32 ± 0.13	802.05 ± 3.36	3.65	0.193	1.883	95.61
	5 × 10^–5^	4.722 ± 1.08	0.8133 ± 0.08	11.11 ± 2.71	894.53 ± 2.07	19.21 ± 0.08	913.74 ± 2.32	3.21	0.215	1.540	96.14
	1 × 10^–4^	4.226 ± 0.95	0.8345 ± 0.07	9.58 ± 2.32	1018.73 ± 4.11	23.25 ± 0.08	1041.98 ± 2.27	2.81	0.266	0.802	96.62
	5 × 10^–4^	7.287 ± 1.21	0.8187 ± 0.11	8.04 ± 1.52	1108.53 ± 3.21	27.08 ± 0.12	1135.61 ± 1.56	1.46	0.293	0.626	96.90
	1 × 10^–3^	7.578 ± 0.86	0.8096 ± 0.09	6.78 ± 1.77	1214.17 ± 2.33	31.37 ± 0.11	1245.54 ± 1.72	0.35	0.303	0.792	97.17

The slow adsorption process of DCSM-8, DCSM-10, and DCSM-12 molecules over *CS* surface^42,85^. From Table 3, at 1 × 10^–3^ M of DCSM-8, DCSM-10, and DCSM-12 inhibitors, *R*_P_ value increased till reach 1004.15 Ω.cm^2^, 1116.56 Ω.cm^2^ and 1245.54 Ω.cm^2^ respectively relative to free 4 M HCl solution 6.55 Ω.cm^2^. this observation indicated the high adsorption power of the prepared inhibitors and their protection role for *CS* in acidic solution^68,86,87^. The calculated value of ${\eta }_{EIS}$ in Table 3 confirmed the mitigation effect of the prepared surfactants via protective insulation film formation covered *CS* surface from the aggressive specie^88^. ${\eta }_{EIS}$ value increased till reach 96.49%, 96.84% and 97.17% at 1 × 10^–3^ M of DCSM-8, DCSM-10, and DCSM-12 inhibitors respectively. This bolstered DCSM-12 with high inhibition efficacy which can be explained by extra *CS* surface coverage and blocking of film pores which enhanced the mitigation potency for *CS*^89–91^. Finally, the goodness of the EIS data is χ^2^ (chi–squared) was measured in the absence and presence of the investigated inhibitors to identify the accurate degrees of EIS data and listed in Table 3. The observed data showed that, χ^2^ of EIS with lower values in both the treated and untreated solutions reflecting that, EIS results were more accurately calculated by utilizing the equivalent circuit model^92,93^. Also, the obtained data of *W*_*L*_, PDP and EIS measurements were in good agreement with each other.

### Weight loss and film stability studies

The inhibition performance of the studied DCSM-8, DCSM-10, and DCSM-12 for *CS* in corrosive surrounding containing 4 M HCl was evaluated using *W*_*L*_ measurements. According to the calculated data in Table 4, after 6 h, the inhibition efficacy of the prepared inhibitors increased with mitigators doses which reflected more *CS* surface covered with surfactants molecules^94^. Also, a decrease in *r* values exhibited *CS* was protected effectively by the introducing surfactants which can be clarified by their high adsorption over *CS* surface via their active sites in their molecular structures^95^. As noticed in Table 4. The mitigation efficacy of the studied surfactants increased till reach 93.537%, 94.003%, and 96.463% for DCSM-8, DCSM-10, and DCSM-12 respectively at 1 × 10^–3^ M. This obtained data confirmed the high defensive role of the prepared surfactants for *CS* in 4 M HCl solution which can be explained by their adsorption over *CS* surface via their active sites as hetero atoms (N and O), π electrons in double bond, + ve nitrogen besides the role of hydrophobic chain^96,97^.

**Table 4:** Weight loss parameters for *CS* in 4.0 M HCl free and containing various concentration of the prepared inhibitors at room temperature.

Inh	Conc. (M)	r, g/cm^2^.h	θ	${\eta }_{{W}_{L}}$
–	Blank	2.98	–	–
DCSM-8	1 × 10^–6^ M 1 × 10^–5^ M 5 × 10^–5^ M 1 × 10^–4^ M 5 × 10^–4^ M 1 × 10^–3^ M	0.4102 0.3382 0.2886 0.2413 0.2077 0.1926	0.86234 0.88651 0.90315 0.91903 0.93031 0.93537	86.234 88.651 90.315 91.903 93.031 93.537
DCSM-10	1 × 10^–6^ M 1 × 10^–5^ M 5 × 10^–5^ M 1 × 10^–4^ M 5 × 10^–4^ M 1 × 10^–3^ M	0.3385 0.3026 0.2653 0.2141 0.1856 0.1787	0.88640 0.89846 0.91097 0.92815 0.93772 0.94003	88.64 89.846 91.097 92.815 93.772 94.003
DCSM-12	1 × 10^–6^ M 1 × 10^–5^ M 5 × 10^–5^ M 1 × 10^–4^ M 5 × 10^–4^ M 1 × 10^–3^ M	0.2817 0.2534 0.2095 0.1719 0.1307 0.1054	0.90546 0.91497 0.92971 0.94232 0.95614 0.96463	90.546 91.497 92.971 94.232 95.614 96.463

The protective film stability of the prepared surfactant was studied at harsh conditions such as different immersion time (6h—24 h) and temperature (25 °C—70°C) using 1 × 10^–3^ M of DCSM-8, DCSM-10, and DCSM-12. The mitigation efficacy in Table 5 of the studied inhibitors increased with exposure time till touch 95.016%, 95.518%, and 97.408% for DCSM-8, DCSM-10, and DCSM-12 respectively after 24 h which can be attributed to their strong adsorption capacity on *CS* surface and formation of insulation defensive layer against the corrosive species^7,29^. While at various temperature, no notable change in the calculated inhibition efficiency in Table 6 for the prepared inhibitors which can be clarified by their strong adsorption over *CS* surface and linking via chemical bond formation^98,99^. All these annotations bolstered formation of stable film layer of the prepared inhibitors molecules against the corrosive particles at harsh conditions^100,101^. Data obtained from *W*_*L*_ measurements showed that, the studied DCSM-12 mitigated *CS* corrosion more than the others mitigators which can be attributed to presence of longer hydrophobic chain in its structure.

**Table 5:** Weight loss parameters for *CS* with time in absence and presence of 1 × 10^–3^ M of prepared inhibitors at room temperature.

*Inh*	6 h			9 h			12 h			24 h		
	*r,* g/cm^2^.h	*θ*	${\eta }_{{W}_{L}}$	*r,* g/cm^2^.h	*θ*	${\eta }_{{W}_{L}}$	*r,* g/cm^2^.h	*θ*	${\eta }_{{W}_{L}}$	*r,* g/cm^2^.h	*θ*	${\eta }_{{W}_{L}}$
Blank	2.98	–	–	4.27	–	–	5.97	–	–	8.19	–	–
DCSM-8	0.1926	0.93537	93.537	0.2374	0.9444	94.440	0.3082	0.94838	94.838	0.4082	0.95016	95.016
DCSM-10	0.1787	0.94003	94.003	0.2261	0.94705	94.705	0.2836	0.95249	95.249	0.3671	0.95518	95.518
DCSM-12	0.1054	0.96463	96.463	0.1361	0.96813	96.813	0.1706	0.97142	97.142	0.2123	0.97408	97.408

**Table 6:** Weight loss parameters for *CS* in absence and presence of 1 × 10^–3^ M of prepared inhibitors at different temperature.

*Inh*	25 ºC			35 ºC			45 ºC			55 ºC		
	*r,* g/cm^2^.h	*θ*	${\eta }_{{W}_{L}}$	*r,* g/cm^2^.h	*θ*	${\eta }_{{W}_{L}}$	*r,* g/cm^2^.h	*θ*	${\eta }_{{W}_{L}}$	*r,* g/cm^2^.h	*θ*	${\eta }_{{W}_{L}}$
Blank	2.98	–	–	4.86	–	–	6.31	–	–	9.47	–	–
DCSM-8	0.1926	0.93537	93.537	0.2986	0.93856	93.856	0.4216	0.93318	93.318	0.6862	0.92753	92.753
DCSM-10	0.1787	0.94003	94.003	0.2773	0.94294	94.294	0.3821	0.93944	93.944	0.6073	0.93587	93.587
DCSM-12	0.1054	0.96463	96.463	0.1684	0.96535	96.535	0.2453	0.96112	96.112	0.4164	0.95603	95.603

**Fig. 8:**
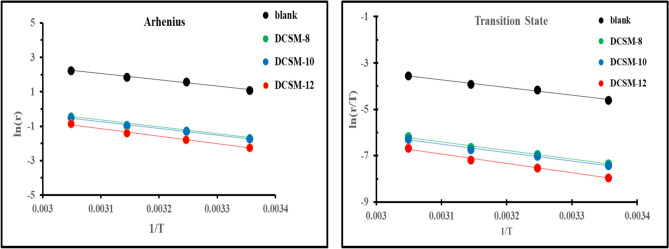
Arrhenius and Transition state plots for *CS* in the absence and presence of prepared DCSM-8, DCSM-10, and DCSM-12 inhibitors at different temperatures.

Also, the activation thermodynamic parameters of *CS* in Table 7 such as E_a_ (activation energy), ΔH* (activation enthalpy) and ΔS* (activation entropy) were determined at temperature range (25 ºC – 55 ºC) based on data obtained from *W*_*L*_ measurements in absences and presence of 1 × 10^–3^ M of the studied DCSM-8, DCSM-10, and DCSM-12 inhibitors according to Arrhenius and Transition state equations:

13 $$\text{ln}r=\text{ln}A-\frac{{E}_{a}}{RT}$$

14 $$\text{ln}\left(\frac{r}{T}\right)=\left[\text{ln}\left(\frac{R}{{N}_{A}h}\right)+\frac{\Delta {S}^{*}}{R}\right]-\frac{\Delta {H}^{*}}{RT}$$

where, A, R, T, N_A_ and h are Arrhenius constant, gas constant, absolute temperature, Avogadro’s number and Plank constant respectively^102^. E_a_ value in Table 7 was calculated based on the slope obtained from ln *r* vs. 1/T relationship as in Fig. 8 The value of *E*_a_ after the addition of the studied mitigators (32.397 kJ.mol^-1^—36.498 kJ.mol^-1^) was very closed to that in 4 M HCl free (30.335 kJ.mol^-1^). The higher activation energy values can be associated with physical adsorption. On the other hand, the adsorption phenomenon of an organic molecule is not considered only as a physical or as a chemical adsorption phenomenon. The slight increase in activation energy after addition the inhibitor can be correlated with the increased thickness of the double layer which enhanced the activation energy of the corrosion process, besides, the competitive adsorption of inhibitors molecules with water molecules whose desorption from the surface needs also some activation energy and formation of inhibitor-Fe complexes which also required additional energy^103–105^. Also, the tabulated ΔH* in Table 7 with positive value for all investigated inhibitors demonstrating that, the activated complex formation during corrosion process is an endothermic reaction. However, the elevation in ΔH* value is markedly accompanied with the existence of the prepared inhibitors which exhibited that, *CS* dissolution process was more difficult after inhibitors addition^35,106^. The obtained ΔS* value with -ve sign bolstered activated complex formation rather than dissociation which reflecting more order occurrence (from reactant to activated complex) accompanied with enhancement in the inhibitor’s efficiency^42,107^.

**Table 7:** Activation thermodynamic parameters of *CS* in 4 M HCl free and containing DCSM-8, DCSM-10, and DCSM-12 at several temperatures.

*Inh*	Arrhenius		Transition state	
	*E*_a,_ (kJ.mol^-1^)	R^2^	ΔH*, (kJ.mol^-1^)	ΔS*, (J.mol^-1^)
Blank	30.335	0.9898	27.738	-142.498
DCSM-8	36.498	0.9939	29.802	-149.962
DCSM-10	33.733	0.9941	31.136	-154.199
DCSM-12	32.397	0.9937	33.901	-159.241

### Adsorption isotherm

**Fig. 9:**
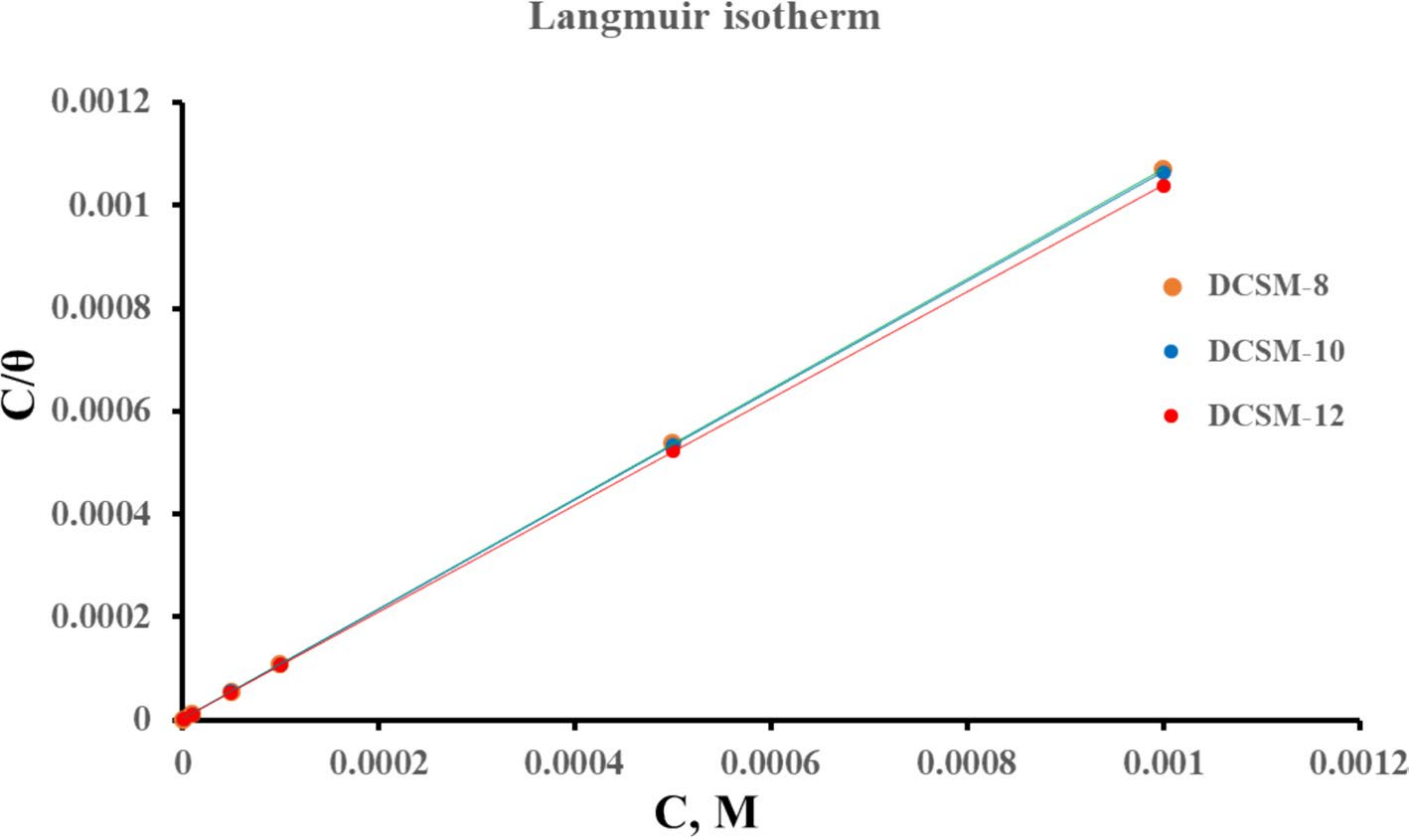
Langmuir adsorption isotherm of the prepared DCSM-8, DCSM-10, and DCSM-12 inhibitors at room temperature.

Based on θ (surface coverage) value obtained from* W*_*L*_ measurements, the adsorption behavior of the prepared DCSM-8, DCSM-10, and DCSM-12 in the aqueous acidic solutions (4 M HCl) was studied using Langmuir adsorption isotherms showing linear relationship of C/θ vs. C (concentration) with slope and correlation coefficient (R^2^) equals to 1.0 as shown in Fig. 9 according to the following equation:

15 $$C / \theta = (1 / {K}_{ads}) + C$$

where, K_ads_ is the equilibrium adsorption constant. The calculated value of K_ads_ in Table 8 exhibited a strong contact between the prepared surfactants and *CS* surface via their active sites as π electrons in double bond, + ve nitrogen and hetero atoms (N and O) with unfilled 3d-orbital of Fe forming a defensive film layer protecting *CS* surface from the corrosive surrounding^108^. DCSM-12 has higher K_ads_ value indicating its superior adsorption capacity in 4 M HCl solution^81,82^. The standard free energy of adsorption (${\Delta G}_{ads}^{o}$) was estimated as follow:

16 $${\Delta G}_{ads}^{o}= - RT ln (55.5 {K}_{ads})$$

where, (55.5) is the molarity of water. The values of ${\Delta G}_{ads}^{o}$ in Table 8 were ranged from—43.459 kJ.mol^-1^ to – 47.177 kJ.mol^-1^ which demonstrated that the prepared DCSM-8, DCSM-10, and DCSM-12 inhibitors were adsorbed on metal surface through chemical adsorption via formation of coordination bonds between lone pairs of electrons on hetero atoms (N and O) and vacant orbitals of iron^43^. Also, ${\Delta G}_{ads}^{o}$ value in Table 8 with -ve sign reveals a spontaneous process that is often characterized by strong contact and a highly effective adsorption^109^^,^^110^.

**Table 8:** Langmuir adsorption isotherm for DCSM-8, DCSM-10, and DCSM-12 at room temperature.

*Inh*	Slope	R^2^	K_ads_, (L.mol^-1^)	$\Delta {G}_{\text{ads}}^{^\circ }$ (kJmol^-1^)
DCSM-8	1.068721	1	747,841.8	-43.459
DCSM-10	1.063195	1	1,012,967	-44.211
DCSM-12	1.060513	1	3,353,075	-47.177

### Quantum chemical study

#### Density functional theory (DFT)

**Fig. 10:**
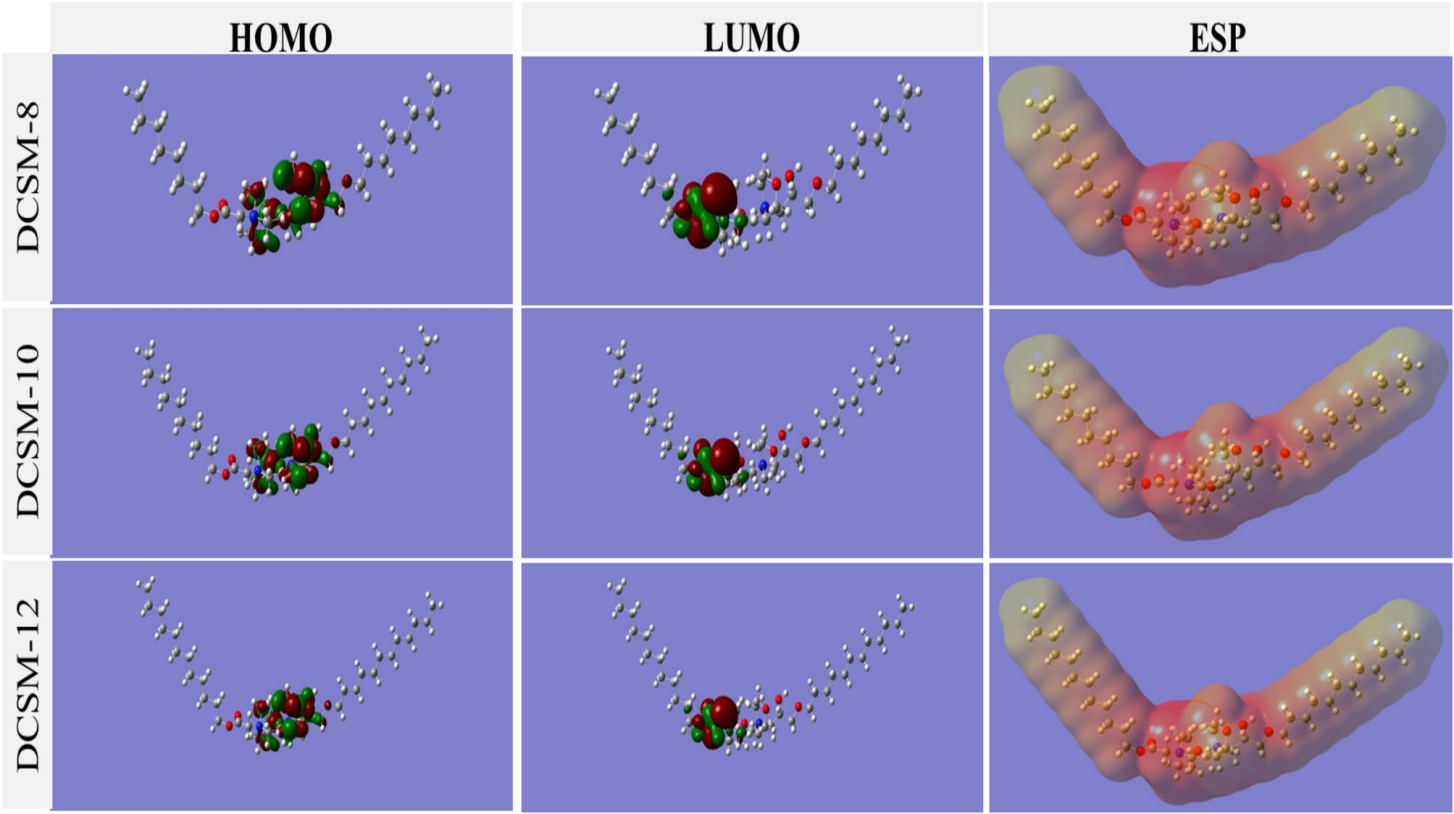
HOMO, LUMO, and ESP of the neutral structures of the studied DCSM-8,

**Fig. 11:**
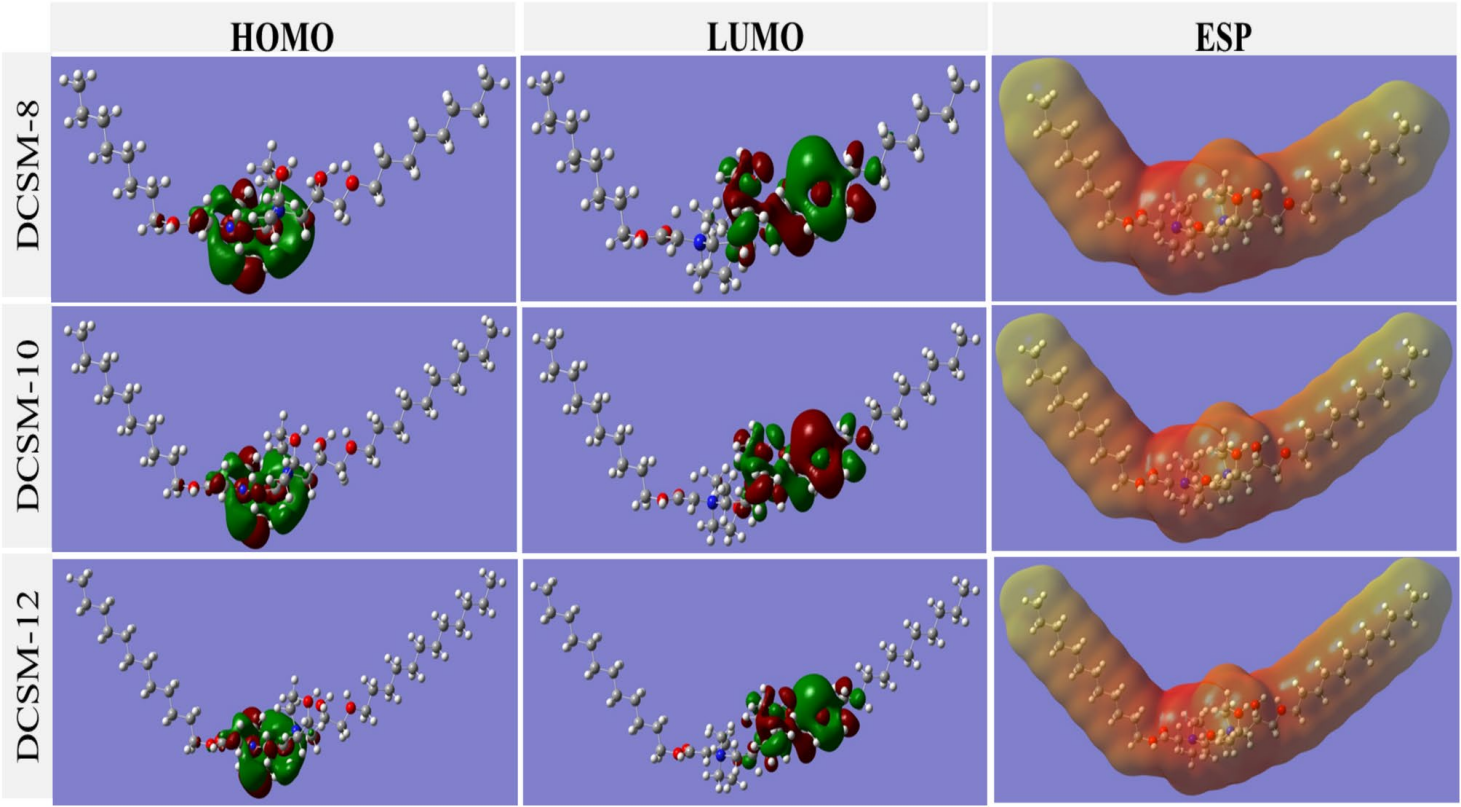
HOMO, LUMO, and ESP of the protonated structures of the studied DCSM-8, DCSM-10, and DCSM-12 using Gaussian 09, Rev. C.01.

After studying the optimized molecular structures of the neutral and protonated structures for prepared inhibitors in Fig. 10 and 11, HOMO region with the highest molecular occupation was localized essentially over the adjacent hydroxyl-ether, quaternary amine group (N^+^) and carbonyl-ether groups, which are responsible for adsorption on the Fe surface through electron donation to the empty d-orbitals. While, LUMO with the least empty molecular orbital represents sites related to electron acceptance from Fe surface through a back-bonding mechanism orbitals^3,28^. The electrostatic potential (ESP) distributed on DCSM-8, DCSM-10, and DCSM-12 active sites attacks, as less-negative yellow areas belong to electrophilic region (poor-electron areas) and the positive red portions belongs to nucleophilic region (electron-rich areas)^87,111^. The HOMO–LUMO gap (∆E) between electron density surfaces, E_HOMO_ and E_LUMO_ are the lowest energy excitation of electrons from HOMO to LUMO which predict the molecules reactivity. ∆E values for the examined inhibitors in Table 9 follows the series: DCSM-12 < DCSM-10 < DCSM-8 which goes along with the experimental measurements, as lower ∆E value correspond to easier electron excitation, giving more electrons to, and adsorb onto *CS* surface^85^. Also, some quantum indices such as *χ*, *η*, and Δ*N* are electronegativity, hardness, and fraction of electron transfer respectively were calculated based on the energies of E_HOMO_ and E_LUMO_ values as the following equations:

17 $$I=-\boldsymbol{ }{E}_{\text{HOMO}}$$

18 $$A=-{E}_{LUMO}$$

19 $$\eta =\frac{\Delta E}{2}$$

20 $$\chi =\frac{-({E}_{HOMO}+{E}_{LUMO})}{2}$$

21 $$\Delta N=\frac{({\varphi }_{Fe}-{\chi }_{Inh})}{[2(\left({\eta }_{Fe}+{\eta }_{Inh.}\right)]}$$

where, I, A, and $\varphi$ are ionization potential, electron affinity, and work function of Fe (1 1 0) plan (= 4.82 eV) respectively^85^. Data in Table 9 clarified that, ∆N value followed the rule of: DCSM-12 > DCSM-10 > DCSM-8, suggesting the electron-sharing between the investigate inhibitors and Fe vacant orbitals that powered the inhibitors molecules adsorption over CS surface, besides the positive value of ΔN reveals the electron-donation capacity of the studied inhibitors towards.

**Table 9:** Quantum chemical parameters of the prepared DCSM-8, DCSM-10, and DCSM-12 inhibitors.

Parameters	Neutral			Protonated		
	DCSM-8	DCSM-10	DCSM-12	DCSM-8	DCSM-10	DCSM-12
E_HOMO_ (eV)	-9.17	-8.31	-6.41	-9.66	-8.78	-6.67
E_LUMO_ (eV)	-3.82	-3.71	-3.51	-3.28	-3.29	-3.57
∆E (eV)	5.35	4.59	2.90	6.38	5.49	3.10
I (eV)	9.17	8.31	6.41	9.66	8.78	6.67
A (eV)	3.82	3.71	3.51	3.28	3.29	3.57
χ (eV)	6.50	6.01	4.96	6.47	6.04	5.12
*η* (eV)	2.68	2.30	1.45	3.19	2.74	1.55
∆N	0.09	0.22	0.70	0.08	0.17	0.61

the vacant 3d-orbitals of Fe which powered the adsorption process of inhibitors molecules over *CS* surface and their anticorrosion property^112^. Also, the previously quantum parameters were also computed for the protonated structures of the studied inhibitors in Table 9 suggested their higher adsorption capacity and their inhibition. Δ*N* values are less than those of the neutral structures which indicated the electron transfer from *CS* surface to the protonated inhibitors molecules. Thus, it can be rationally inferred that the neutral inhibitor molecules generate mainly donating electrons to the empty 3*d* orbital of the iron atoms while the protonated ones have stronger capability of electron-acceptance from the iron atoms, hence strengthening^77^. In general, the quantum calculations showed the ability of DCSM-8, DCSM-10, and DCSM-12 molecules to shield *CS* surface through their adsorption via electrons transfer from the HOMO centered on the oxygen and nitrogen atoms in the middle of the inhibitors structure to *CS* empty d-orbitals besides the back donation of electrons from metal surface to LUMO regions in DCSM-8, DCSM-10, and DCSM-12 structures^85,113^.

#### Monte carlo simulations (MCs)

**Fig. 12:**
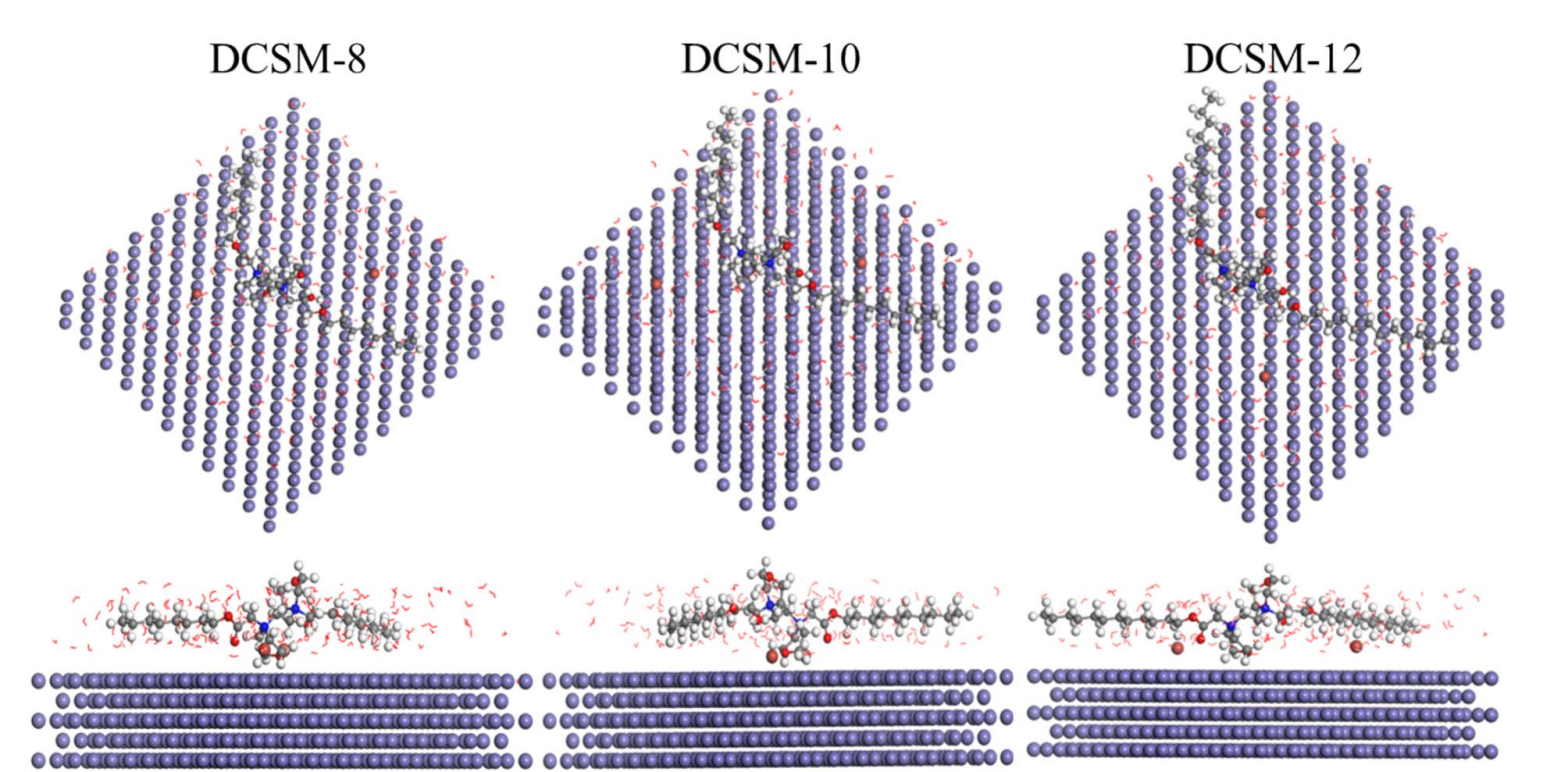
Equilibrium adsorption configuration of the studied DCSM-8, DCSM-10, and DCSM-12 on Fe (110) obtained by MCs simulations using BIOVIA Materials Studio 6.0 (17.1.0.48).

The adsorption behavior of the prepared DCSM-8, DCSM-10, and DCSM-12 on Fe (110) surface was studied using MCs simulations as shown in Fig. 12 providing their equilibrium configuration. Inspection of Fig. 12 reveals that the prepared inhibitors adsorbed on Fe (110) surface horizontally with a flat orientation reflecting high *CS* surface coverage against the corrosive surrounding. The adsorption energy (E_ads_) values in Table 10 for the studied surfactants based on MCs showed that, their E_ads_ value was very low compared with that of corrosive species confirming the stronger adsorption ability of prepared inhibitors via replacement process of water molecules with inhibitors molecules and formation of an adsorption layer over *CS* surface^114–118^. Also, DCSM-12 with the lowest E_ads_ value exhibited its superior mitigation potency for *CS* than DCSM-8 and DCSM-10. As noticed, -ve value of E_ads_ for the investigated inhibitors in Table 10 bolstered their spontaneous nature and strong adsorption on *CS* surface^112^. The mitigation role of the prepared inhibitors increases with alkyl chain increases via forcing inhibitors molecules to cover and protect extra *CS* surface area against the corrosive particles^9,10^. Finally, the theoretical studies of DCSM-8, DCSM-10, and DCSM-12 confirmed their mitigation performance and supported the experimental results.

**Table 10:** The outputs energies calculated by MCs for DCSM-8, DCSM-10, and DCSM-12 on Fe (110).

System	Total energy (kcal/mol)	Adsorption energy (kcal/mol)	Rigid adsorption energy (kcal/mol)	Deformation energy (kcal/mol)	dE_ads_/dN_i_ (kcal/mol)	dE_ads_/dN_i_ of H_2_O (kcal/mol)
Fe + H_2_O + DCSM-8	66.58	-118.16	-9.77	-108.39	-114.79	-3.08
Fe + H_2_O + DCSM-10	67.50	-120.75	-11.78	-108.98	-117.28	-3.36
Fe + H_2_O + DCSM-12	67.84	-123.93	-14.56	-109.36	-120.56	-3.42

### Surface analysis (SEM and EDX)

**Fig. 13:**
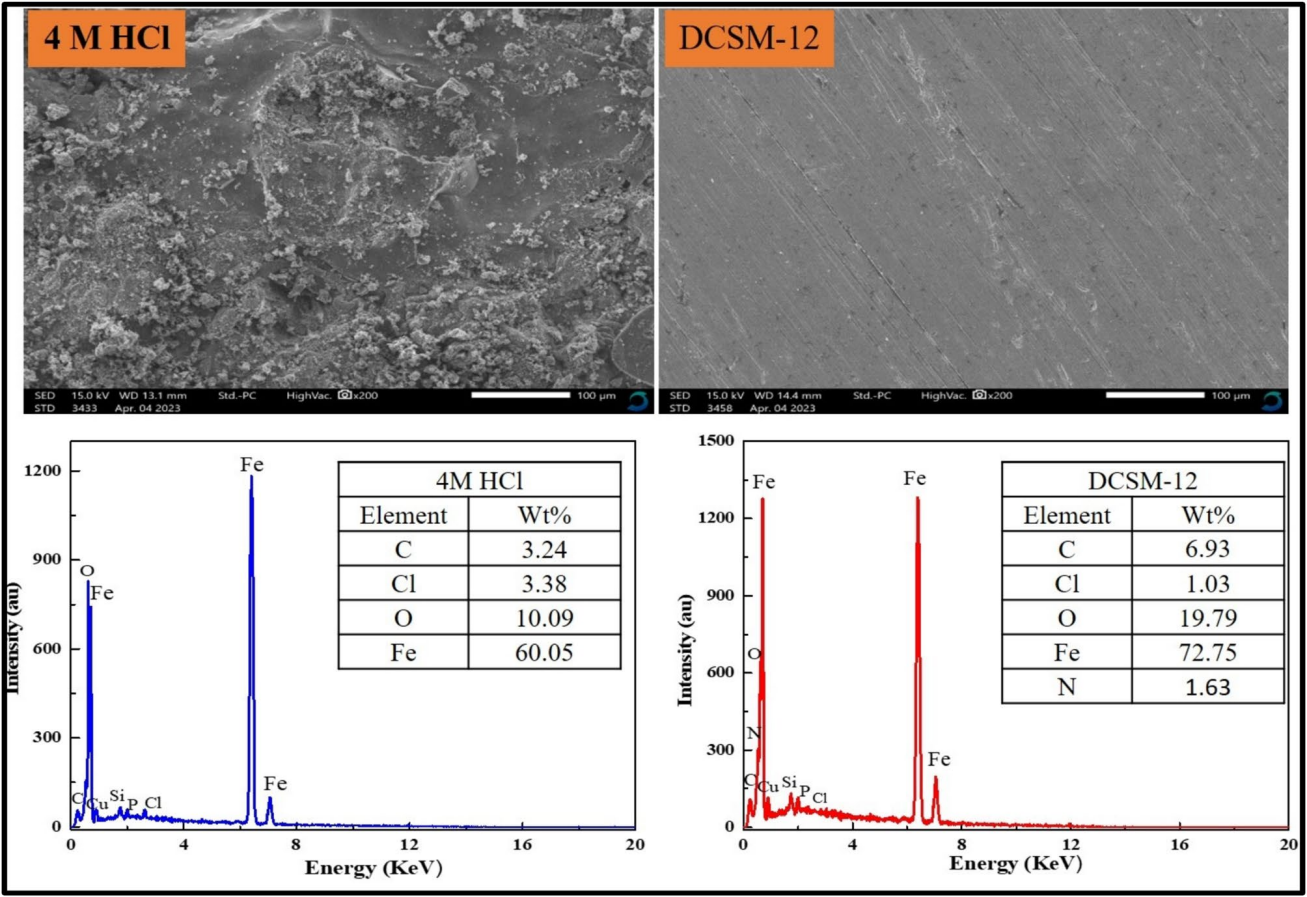
SEM and EDX for *CS* in 4 M HCl free and after addition of 1 × 10^–3^ M of DCSM-12 inhibitor.

*CS* surface morphology investigations were studied using SEM as seen in Fig. 13 showing *CS* 2D images in 4 M HCl solution in absence and presence 1 × 10^–3^ M of the studied DCSM-12. *CS* image in 4 M HCl free exhibited roughly and damaged *CS* surface which can be explained by the destructive effect of the acidic solution^82,119^. While after treated with DCSM-12 inhibitor, *CS* surface became more smooth and homogenous owing to insulation defensive layer formation over *CS* surface that decreased the HCl/*CS* contact^110^. EDX spectra in Fig. 13 give a quantitative data about *CS* outer layer in 4 M HCl free with and without 1 × 10^–3^ M of the investigated DCSM-12. The weight percentage value of Fe, C, O, Cl^-^ and N atoms confirms SEM annotations. Fe and C peaks intensities increase while O and Cl^-^ peaks intensity decreases after the addition of DCSM-12 inhibitor. Also, the appearance of N-peak confirmed the existence of the DCSM-12 molecules over *CS* surface forming a protective film layer^120^. These annotations confirmed the blocking power of the prepared DCSM-12 and its role in *CS* protection via construction of an insulation defensive layer of DCSM-12 molecule over *CS* surface against the destructive particles^96^.

## Conclusion

Three novel Di-cationic surfactants based on morpholinium have been conducted successfully and characterized by FT-IR, HNMR, and mass analysis and evaluated as corrosion mitigators for *CS* in 4 M HCl solution via chemical (*W*_*L*_) and electrochemical techniques (PDP and EIS). Surface activity measurements showed their high ability towards surface tension reduction with low CMC. The application of the prepared surfactants showed an excellent inhibition effect against *CS* corrosion in 4 M HCl with inhibition efficacy 97.029% for DCSM-12. The adsorption of the synthesized surfactants over *CS* surface followed Langmuir adsorption isotherm via chemical adsorption. The quantum computations and the MC simulations were found in a good agreement with the obtained experimental data. Finally, SEM and EDX analysis showed a significant improvement in *CS* surface morphology with the existence of DCSM-12 which confirmed DCSM-12 adsorption capacity.

## Supplementary Information

Supplementary Information.

## Data Availability

All data generated or analyzed during this study are included in this manuscript.
