# Supplementary material for: Multidimensional insights of electrochemical and quantum investigations of morpholinium cationic surfactants as corrosion inhibitors for carbon steel in acidic solution
Source: Sci Rep. 2025 Jun 20;15:20175. doi: 10.1038/s41598-025-05836-x (PMC12181321; doi:10.1038/s41598-025-05836-x)
Supplement: Supplementary file 1 — Supplementary Information. [file 41598_2025_5836_MOESM1_ESM.docx]

Fig.1Si: Synthesis of cationic surfactants.

Table.1Si: Standard deviation values of *R*_P_ and *i*_corr_ for the *CS* in absence and presence of the prepared inhibitors.

| ***Inh.*** | **Conc. M** | ***i*_corr_,**  **mA** | | ***R*_P_,** (Ω.cm^2^) | |
| --- | --- | --- | --- | --- | --- |
|  |  | value | *Sd* | value | *Sd* |
| ***----*** | Blank | 23.60 | 0.80829 | 35.24 | 3.65044 |
| **DCSM-8** | 1×10^-6^ | 2.812 | 0.54720 | 553.77 | 3.74189 |
|  | 1×10^-5^ | 2.394 | 0.46736 | 637.95 | 3.44353 |
|  | 5×10^-5^ | 2.057 | 0.46177 | 716.76 | 2.65975 |
|  | 1×10^-4^ | 1.891 | 0.59050 | 848.63 | 2.50096 |
|  | 5×10^-4^ | 1.684 | 0.62661 | 911.31 | 3.56375 |
|  | 1×10^-3^ | 1.424 | 0.44057 | 1004.15 | 3.36402 |
| **DCSM-10** | 1×10^-6^ | 2.727 | 0.60105 | 602.43 | 3.33144 |
|  | 1×10^-5^ | 2.254 | 0.55105 | 695.82 | 2.49044 |
|  | 5×10^-5^ | 2.002 | 0.38180 | 785.56 | 3.14575 |
|  | 1×10^-4^ | 1.791 | 0.51632 | 892.58 | 1.64508 |
|  | 5×10^-4^ | 1.558 | 0.45574 | 988.13 | 1.86937 |
|  | 1×10^-3^ | 1.309 | 0.43448 | 1116.56 | 2.03663 |
| **DCSM-12** | 1×10^-6^ | 2.286 | 0.42861 | 687.03 | 2.46792 |
|  | 1×10^-5^ | 2.041 | 0.20368 | 802.05 | 2.46736 |
|  | 5×10^-5^ | 1.595 | 0.24686 | 913.74 | 2.95020 |
|  | 1×10^-4^ | 1.112 | 0.19416 | 1041.98 | 1.99481 |
|  | 5×10^-4^ | 0.902 | 0.18093 | 1135.61 | 3.05352 |
|  | 1×10^-3^ | 0.701 | 0.22755 | 1245.54 | 1.92174 |


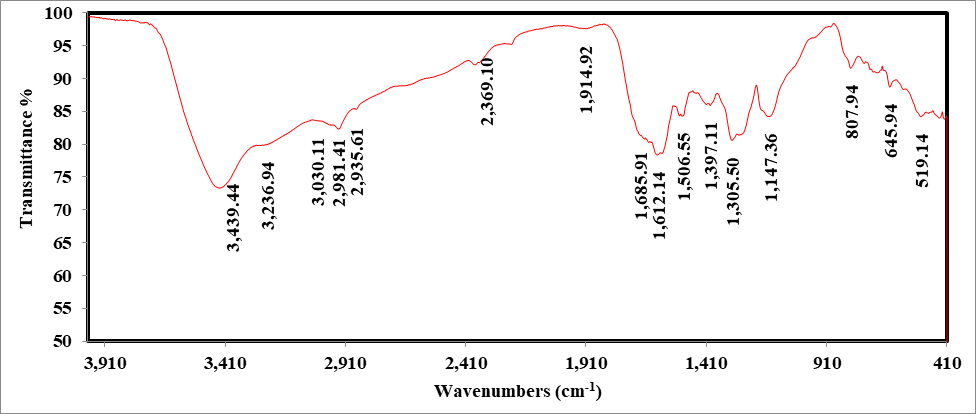

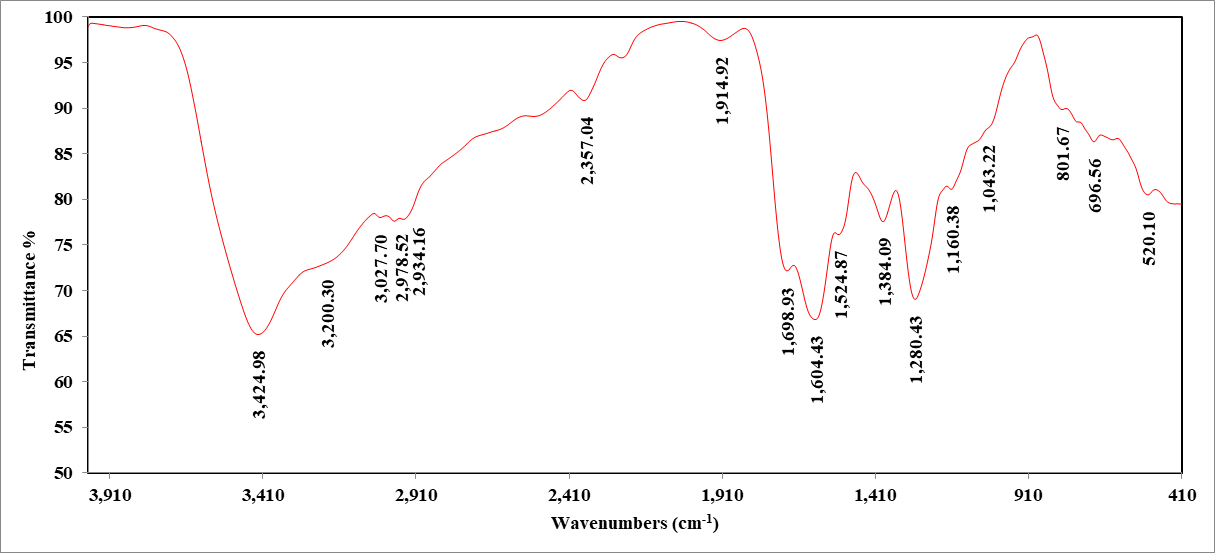


DCSM-8

DCSM-10

Fig.2Si: FT-IR of the prepared DCSM-8 and DCSM-10.


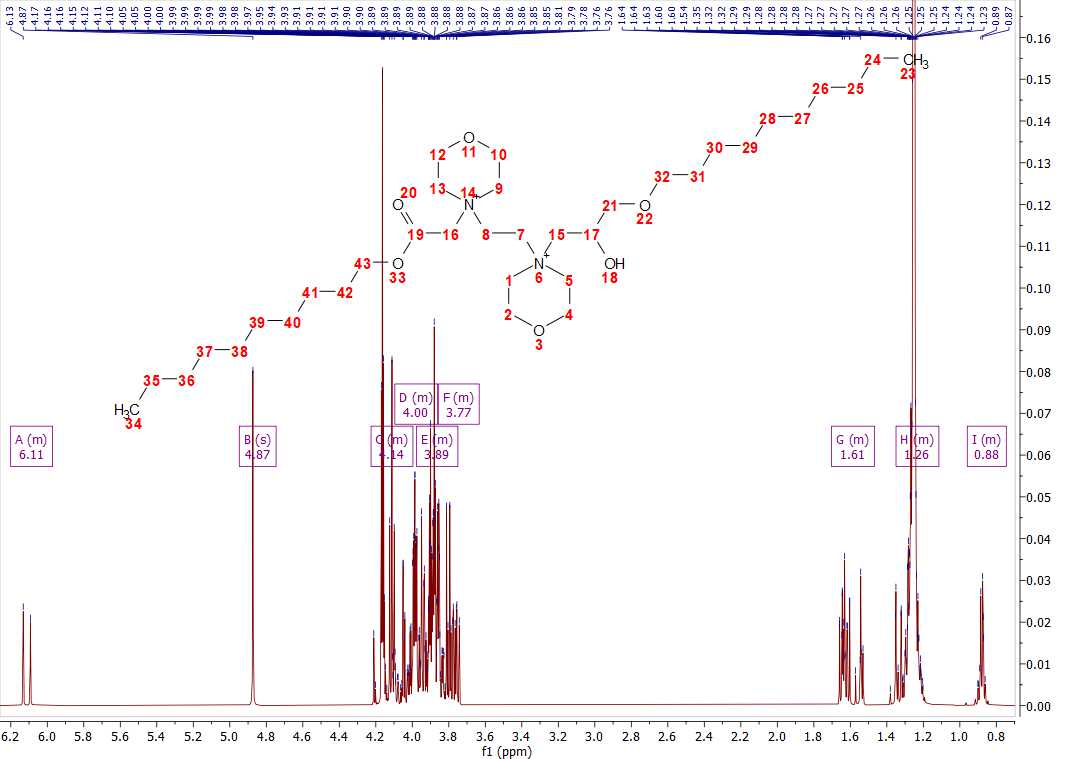

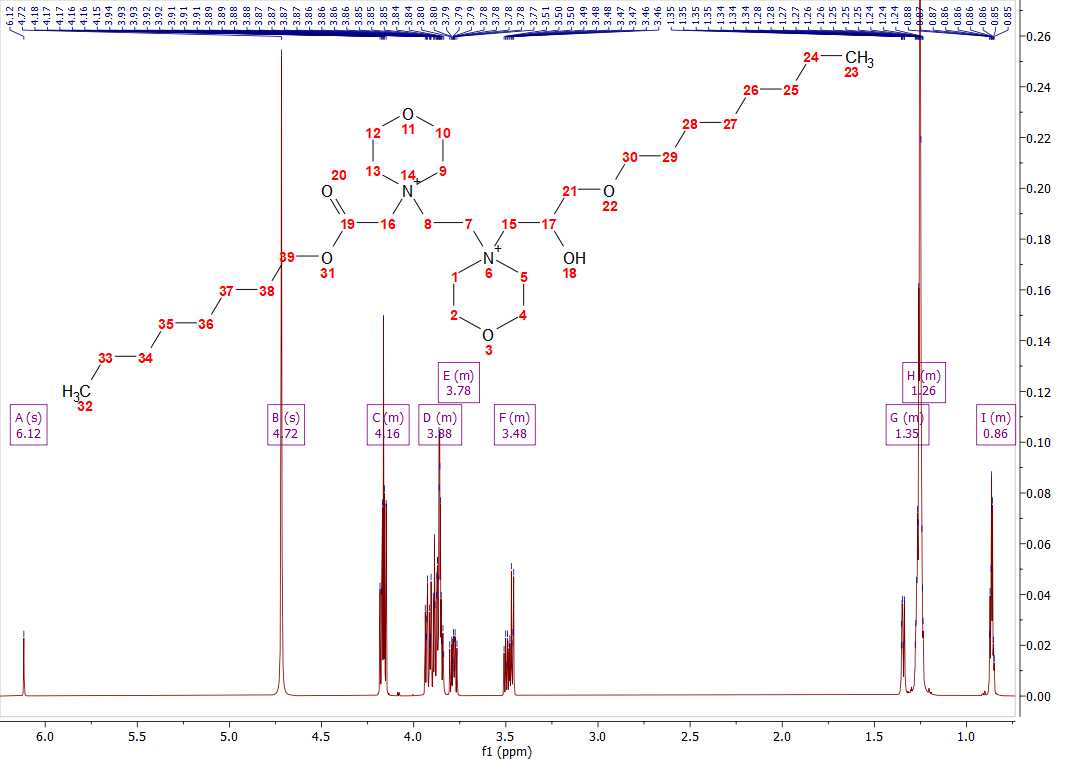


Fig.3Si: ^1^HNMR of the prepared DCSM-8 and DCSM-10.

Fig.4Si: Mass spectra of the prepared DCSM-8 and DCSM-10.

DCSM-8

DCSM-10


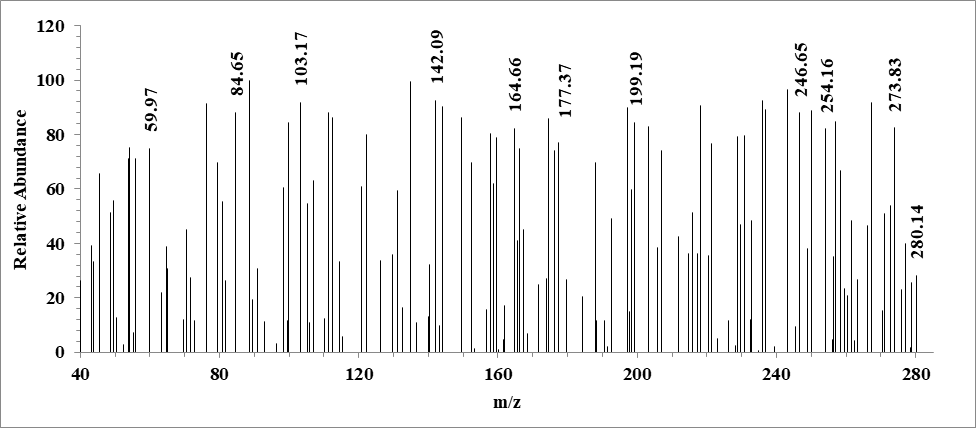

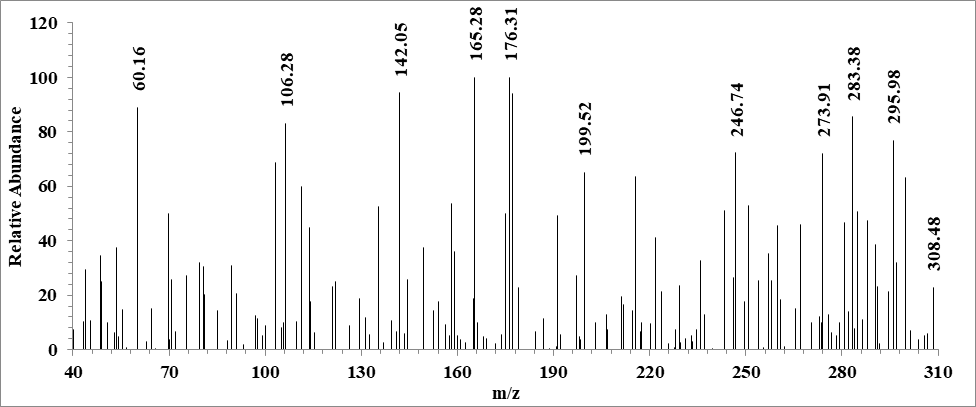


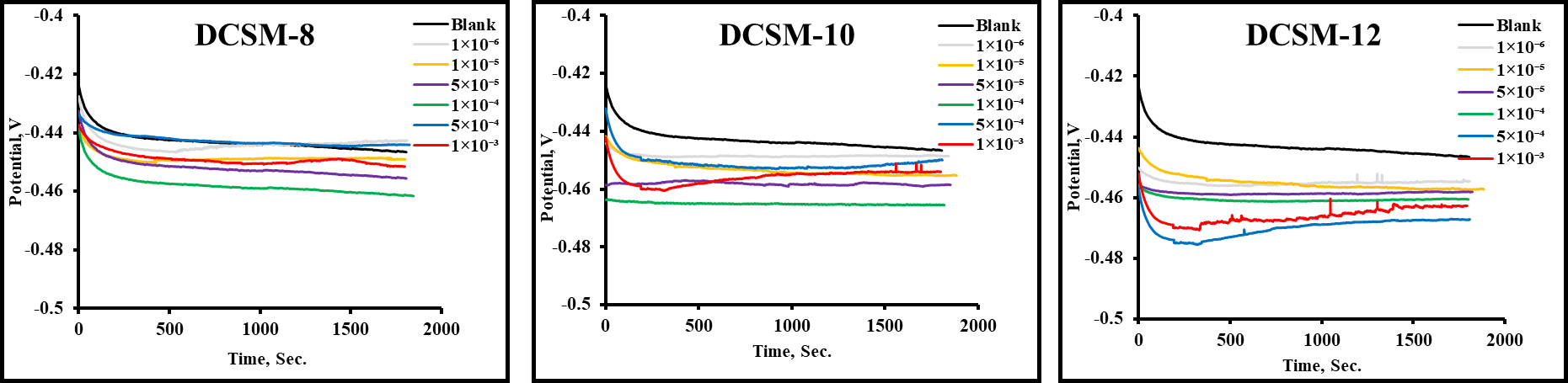


Fig.5Si: OCP Vs Time for *CS* in 4 M HCl in absence and presence of different concentrations of the prepared inhibitors at room temperature.
